# Identification and Analysis of the Role of Superoxide Dismutases Isoforms in the Pathogenesis of *Paracoccidioides* spp.

**DOI:** 10.1371/journal.pntd.0004481

**Published:** 2016-03-10

**Authors:** Diana Tamayo, José F. Muñoz, Ángela Lopez, Martha Urán, Juan Herrera, Clayton L. Borges, Ángela Restrepo, Celia M. Soares, Carlos P. Taborda, Agostinho J. Almeida, Juan G. McEwen, Orville Hernández

**Affiliations:** 1 Cellular and Molecular Biology Unit, Corporación para Investigaciones Biológicas, Medellín, Colombia; 2 Institute of Biology, Universidad de Antioquia, Medellín, Colombia; 3 Institute of Biomedical Science; Department of Microbiology; Universidade do São Paulo; São Paulo, Brazil; 4 Laboratório de Biologia Molecular, Instituto de Ciências Biológicas, ICBII, Goiânia, Brazil; 5 Department of Biological Sciences, School of Sciences, Universidad Eafit, Medellín, Colombia; 6 School of Medicine, Universidad de Antioquia, Medellín, Colombia; 7 MICROBA research group, School of Microbiology, Universidad de Antioquia, Medellín, Colombia; University of Tennessee, UNITED STATES

## Abstract

The ability of *Paracoccidioides* to defend itself against reactive oxygen species (ROS) produced by host effector cells is a prerequisite to survive. To counteract these radicals, *Paracoccidioides* expresses, among different antioxidant enzymes, superoxide dismutases (SODs). In this study, we identified six SODs isoforms encoded by the *Paracoccidioides* genome. We determined gene expression levels of representative isolates of the phylogenetic lineages of *Paracoccidioides* spp. (S1, PS2, PS3 and *Pb01-like*) using quantitative RT-PCR. Assays were carried out to analyze *SOD* gene expression of yeast cells, mycelia cells, the mycelia-to-yeast transition and the yeast-to-mycelia germination, as well as under treatment with oxidative agents and during interaction with phagocytic cells. We observed an increased expression of *PbSOD1* and *PbSOD3* during the transition process, exposure to oxidative agents and interaction with phagocytic cells, suggesting that these proteins could assist in combating the superoxide radicals generated during the host-pathogen interaction. Using *PbSOD1* and *PbSOD3* knockdown strains we showed these genes are involved in the response of the fungus against host effector cells, particularly the oxidative stress response, and in a mouse model of infection. Protein sequence analysis together with functional analysis of knockdown strains seem to suggest that *PbSOD3* expression is linked with a pronounced extracellular activity while *PbSOD1* seems more related to intracellular requirements of the fungus. Altogether, our data suggests that *P*. *brasiliensis* actively responds to the radicals generated endogenously during metabolism and counteracts the oxidative burst of immune cells by inducing the expression of SOD isoforms.

## Author Summary

Paracoccidioidomycosis is a health-threatening human systemic mycosis, endemic to some Latin America countries. The disease is caused by species belonging to the *Paracoccidioides* genus. Once inside the human host, *Paracoccidioides* must face the host innate immune system, escaping from the cytotoxic capacity of innate immune cells (ROS production and liberation of polypeptide antibiotics). To do so, they express and synthetize superoxide dismutases (SODs). We aimed to identify and characterize the SOD isoforms present in the *Paracoccidioides* genome. We identified six isoforms, among which we found an increased expression of *PbSOD1* and *PbSOD3* during the transition-to-yeast process, exposure to oxidative agents and interaction with phagocytic cells. Additionally, we found that *PbSOD3* expression might be linked with a pronounced extracellular activity while *PbSOD1* and the other isoforms seem more related to intracellular requirements of the fungus. We propose that the defence against endogenous-produced ROS may depend on intracellular Sods (mostly *SOD1*, but possibly also *SOD2*, *SOD4* and *SOD5*), but defence against extracellular ROS (produced during host-pathogen interactions) might rely to a greater extent on *SOD3*, which is endowed with an extracellular activity.

## Introduction

Dimorphic fungal pathogens are exposed to reactive oxygen species (ROS) from both internal and external sources. ROS include superoxide anion (O_2_^**.-**^), hydroxyl radical (^.^OH), and hydrogen peroxide (H_2_O_2_), among others. The superoxide anion radical is the first product of oxygen reduction. This radical is mediated by a variety of electron carriers [1] and it is considered the precursor of most other ROS [2]. Internally, ROS are mostly produced in fungal mitochondria as a by-product of aerobic cellular respiration [3]; externally, reactive oxygen and nitrogen species (ROS/RNS) can be produced by host cells during fungal infections [4]. The latter represent an important line of defense and one of the primary effector mechanisms of the host’s immune system aimed at controlling fungal infections [5,6]. At high concentrations, ROS can be extremely harmful at levels that exceed the defense mechanism of the fungal cell, generating an oxidative-stress state that can lead to oxidation of proteins, lipids and DNA, and ultimately to cell death [7]. However, at low concentrations ROS also function as critical second messengers in a variety of intracellular signaling and regulation pathways [8], and have also been correlated with life-span regulation and cell differentiation in microbial eukaryotes [9–12]. Understanding this dual role/effect of ROS is important when defining the components of the fungal antioxidant response and trying to understand the cellular and molecular changes required for adaption to these internal or external stimuli.

To counteract these reactive species, fungal pathogens, such as those belonging to the *Paracoccidioides* genus, are equipped with an antioxidant system that prevents ROS-damaging effects. This antioxidant system includes enzymes such as catalases, peroxidases and superoxide dismutases (SODs) [13]. Additionally, certain metabolic pathways are set in motion in order to supply reducing power, such as the pentose phosphate pathway and the thioredoxin and glutathione redox systems [1,13].

Thermally dimorphic fungi belonging to the genus *Paracoccidioides* are the etiological agents of paracoccidioidomycosis (PCM), a neglected health-threatening human systemic mycosis endemic to Latin America where up to ten million people appear to be infected. The fungus is thought to exist in nature in the mycelial form at environmental temperatures, while within the human host or at 37°C, it grows as the yeast form [14,15]. *Paracoccidioides* species belong to the largest group of dimorphic fungal pathogens, which includes species from the genus *Histoplasma*, *Blastomyces* and *Coccidioides* in the order Onygenales. Within the *Paracoccidioides* genus, there are four well-characterized phylogenetic lineages, all of them capable of infecting humans and causing PCM: three *P*. *brasiliensis* lineages (S1, PS2 and PS3) and one *P*. *lutzii* lineage (Pb01-like) [16,17].

As other fungal pathogens, *Paracoccidioides* spp. are exposed to different aggressions once inside the human host. The higher temperature within the human host has been shown to induce an increase in fungal metabolism leading to a greater oxygen consumption and ROS production in fungi such as *Cryptococcus neoformans*, *Saccharomyces cerevisiae* and *Schizosaccharomyces pombe* [9,18,19]. In addition, *Paracoccidioides* will also encounter host effector cells, which produce ROS during the oxidative burst in the phagolysosome through the activation of the NADPH-oxidase complex, generating superoxide radical as the first intermediate product [20]. *Paracoccidioides* yeast cells cope with these radicals within the phagocytes through the expression of proteins from the antioxidant system, such as SOD enzymes, to neutralize superoxide radicals and convert them into less damaging molecules, namely hydrogen peroxide and oxygen molecules [13]. SODs are metallo-proteins, which are classified on the basis of the metals located in their active sites (Fe, Mn, Ni and Cu/Zn) [21–23]. These proteins have been shown to contribute to the virulence of some pathogenic fungi, namely *Candida albicans* [24,25], *C*. *neoformans* [26], *Aspergillus fumigatus* [27], and *H*. *capsulatum* [20], all of which are capable, to a certain extent, of neutralizing the toxic levels of ROS generated by the host. However, the mechanism by which SOD contributes to the defensive mechanism of *Paracoccidioides* against exogenous and endogenous oxidative damage remains elusive.

In the present study, we sought to identify and characterize the SOD isoforms encoded by the *Paracoccidioides’* genome, with the purpose of better understanding their function during the morpho-physiological transformation inherent to its dimorphic life cycle as well as during host-pathogen interactions. To pursue these goals, we first identified SOD proteins encoded by the *Paracoccidioides* spp. genome and determined gene expression of one representative isolate of each one of the phylogenetic lineages of *Paracoccidioides* spp. Assays were carried out to analyze SODs gene expression of yeast, mycelia cells and conidia undergoing the transition (mycelia-yeast and conidia-yeast) and the germination (yeast-mycelia and conidia-mycelia) processes, as well as under treatment with oxidative agents and during interaction with phagocytic cells (*e*.*g*. human PMNs and alveolar macrophages). Antisense RNA technology was also employed to further evaluate the role of specific isoforms upon exposure to oxidative stress-inducing agents, interaction of yeast cells with phagocytic cells and in a mouse model of infection.

## Materials and Methods

### Identification and sequence analyses of SOD isoforms

In order to identify the SOD isoforms encoded be the *Paracoccidioides* genome and classify its SOD’s orthologs, we used bioinformatics tools based on similarity and orthology analyses. We used bidirectional BLAST analysis version 2.2.28+ with default parameters to identify sequence similarities [28]. Orthology analysis was performed using OrthoMCL version 1.4 with a Markov inflation index of 1.5 and a maximum e-value of 1e-5 [29].

We studied one strain representing each of the lineages of *Paracoccidioides*. Sequences of Pb18, Pb03 and Pb01 strains were chosen to represent S1, PS2 and *Pb01-like* lineages, respectively [30,31]. For the PS3 lineage, we used paired-end reads and reference assembly of the Pb60855 strain (ATCC60855; PbWT60855; ongoing genomic project). For all the identified SOD isoforms, protein domain conservation analyses were done using InterProScan [32], by sequence comparison with InterPro collection of protein signature databases in the EMBL-EBI (http://www.ebi.ac.uk/interpro/). Multiple sequence alignments were constructed using Muscle [33], and phylogenetic trees were constructed employing a distance computation method (Neighbor joining) [34]. Other features in the gene/protein sequences and annotations of the SOD isoforms, were identified using SignalP 4.1 server (http://www.cbs.dtu.dk/services/SignalP/) [35], TargetP 1.1 server (http://www.cbs.dtu.dk/services/TargetP/) [36] and PredGPI predictor (http://gpcr.biocomp.unibo.it/predgpi/pred.htm).

### Microorganisms and growth conditions

A representative isolate from each one of the phylogenetic lineages of *Paracoccidioides* spp. was used. *P*. *brasiliensis* knockdown strains used in this study were derived from the wild-type strain Pb60855. Strains and isolates used in this study are listed in Table 1. *Paracoccidioides* cells were maintained by sub-culturing in brain heart infusion (BHI) supplemented with 1% glucose (Beckton Dickinson and Company, Sparks, MD), under constant agitation at 36°C for the yeast form, and at 20°C for the mycelia form, unless otherwise indicated. *P*. *brasiliensis* conidia were produced and purified using the glass-wool filtration protocol, as described previously by Restrepo et al. [37]. In order to induce and evaluate the transition processes (conidia to yeast (C-Y); mycelia to yeast (M-Y)) and the germination process (yeast to mycelia (Y-M)), cells were incubated at 36°C or at 20°C, respectively, under constant agitation in a flask containing BHI [38,39]. During the morphological switch several fungal morphotypes coexist. The different stages of the dimorphic transition are fully established (for more information see Nunes et. al, 2005 and Hernández et. al 2011). Cultures during M-Y transition are characterized by the presence of hyphae, differentiating hyphae (chlamydospore-like cells), transforming yeast (production of multiple buds by the chlamydospore) and mature, multibudding yeast [40]. During C-Y transition, cultures are characterized by the presence of conidia, intermediate cells and yeast cells. After 12 h, intermediate cells are present, from 48 h onwards it is possible to observe the characteristic yeast cells, although, they are more abundant at 72 h. Regarding to the C-M germination, conidia began to germinate approximately at 24 h and the formation of branched mycelia occurs after 96 h [41,42]. Samples were collected for RNA extraction and quantification of gene expression analyses during evaluated time points.

**Table 1:** Isolates used in this study.

Species	Isolate/strain designation	Phylogenetic lineage
*P*. *brasiliensis*	Pb18	S1
*P*. *brasiliensis*	Pb03	PS2
*P*. *brasiliensis*	Pb60855, PbBAC	PS3
*P*. *lutzii*	Pb01	*Pb01*-like
*P*. *brasiliensis*	PbEV*	PS3
*P*. *brasiliensis*	PbSOD1-aRNA*^	PS3
*P*. *brasiliensis*	PbSOD3-aRNA*	PS3
*A*. *tumefaciens*	LBA1100	N/A
*E*. *coli*	DH5α	N/A

* Background strain Pb60855

^ do Carmo Silva 2015

Samples were collected for RNA extraction and quantification of gene expression analyses during evaluated time points.

*Agrobacterium tumefaciens* strain LBA1100 [43] was used as the recipient for the binary vectors constructed in this study (Table 1). Bacterial cells were maintained at 28°C in Luria–Bertani (LB) medium containing kanamycin (100 mg/ml). *Escherichia coli* DH5α was grown at 37°C in LB medium supplemented with appropriate antibiotics and used as the host for plasmid amplification and cloning.

### Gene expression levels via real-time RT-qPCR analyses

Total RNA was obtained from *Paracoccidioides* cells using the Trizol reagent (Invitrogen). Total RNA was treated with DNase I (Thermo Scientific) and tested using a conventional PCR with β-tubulin primers to confirm the absence of chromosomal DNA contamination. cDNA was synthesized using 2 μg of total RNA with Maxima First Strand cDNA synthesis kit for RT-qPCR, according to the manufacturer’s instructions (Fermentas).

Real-time PCR was carried out using a Maxima SYBR Green/Fluorescein qPCR Master Mix (2X; qRT-PCR) kit with SYBR green, according to the manufacturer’s instructions (Fermentas). The CFX96 real time PCR detection system (Bio-Rad, Hercules, CA) was used to measure gene expression level of SOD isoforms present in the four phylogenetic lineages (S1, PS2, PS3 and *Pb01*-like) encoded into the *Paracoccidioides’* genome. Primers were designed in order to anneal properly to each SOD transcript of the four phylogenetic lineages (S1 Table). Additionally, in Pb60855, SOD isoforms were evaluated in cells undergoing the transition (C-Y and M-Y) and germination processes (C-M and Y-M). We also evaluated gene expression in knockdown strains for *PbSOD1* and *PbSOD3* genes, and in *P*. *brasiliensis* cells carrying the empty binary vector as a control (PbEV60855). Melting curve analysis was performed after the amplification phase to eliminate the possibility of non-specific amplification or primer-dimer formation. β-tubulin gene (housekeeping gene) was used in order to normalize the expression value of each SOD isoform. Each experiment was done in triplicate, and the expression level was measured three times. We also compared the elongation factor 3 (*TEF3*, PABG_05066) as normalizer for the expression experiments. We saw no differences by using *TUBE3* or *TEF3* as normalizers. Accordingly, the calibrator gene used along the expression experiments was the *TUBE3* gene (S1 Fig).

### *P*. *brasiliensis* cellular extracts preparation and Sod zymograms

Yeast cell samples of Pb60855 were collected at 48 hours of growth for protein extraction. Briefly, after a 3000 rpm centrifugation during 15 minutes, the supernatant was collected and subsequently concentrated through a dialysis membrane (Spectra/Por 1, SPECTRUM. MWCO 6–8,000). Cells were washed twice in PBS, then resuspended in 2 ml of 0.05 M Tris-HCl pH 8.5, cocktail protease inhibitors and disrupted using glass beads (0.5 mm diameter) through vortexing. The cell wall fraction was removed by low speed centrifugation (3000 g, 5 min at 4°C) [27], and incubated in a 50 mM NaOAc and 5 mM NaN3 pH 5.6 solution at 36°C under agitation for 12 h. Protein contents of the extracellular, cell wall and intracellular (cytoplasmic) fractions were quantified with the Bradford reagent (Bio-Rad) [44], using bovine serum albumin as the standard and stored at -20°C.

To detect Sod enzymatic activity, protein samples were separated by 9% native acrylamide gel electrophoresis (1D-PAGE; running buffer: 0.025 Tris-HCl, 0.192 M Glycin, pH 8.3), without SDS to keep the proteins activity. Electrophoresis was carried out at 120 V at 4°C. Sod enzymatic activity was visualized as the inhibition of the reduction of nitro blue tetrazolium (NBT; Sigma) according to the method of Beauchamp and Fridovich (1971) [45]. Here, two reactions occur, the first one is the autoxidation from riboflavin and the second one is the riboflavin/NBT reduction, using NBT as chromogenic substrate. The Sod activity is determined as an achromatic zone, since the enzyme inhibits NBT reduction [45]. Following electrophoresis, the gel is washed twice during 10 min on ice-cold water, soaked in 2.45 mM NBT solution for 20 min in the darkness. This was followed by a further incubation in 0.028 mM riboflavin and 0.028 M tetramethylethylenediamine (TEMED) in PBS 1X, pH 7.8, for 15 min in the darkness. Upon illumination, an achromatic band indicating zones of activity appear in the region of gel where Sod proteins are present. For the 2D-PAGE, we followed the method described by Niyomploy *et al*. [46], with minor modifications. Briefly, cytoplasmic crude extracts were loaded onto 7-cm, pH 3–10 IPG gel strips (Bio-RAD), and left overnight at room temperature for the rehydration process. The isoelectrofocusing [47] was performed as described by Niyomploy *et al*.; thereafter, strips were rinsed in running buffer, equilibrated in equilibration buffer (0.05 M Tris-HCl pH6.8, 30% glycerol) for 10 min at room temperature, following by a rinse with running buffer, and incubation with equilibration buffer containing 2.5% iodoacetamide (IAA). Rinsed once again with running buffer and proceeding with the second dimension and zymogram as described above.

### Interaction of *P*. *brasiliensis* yeast cells with host cells

For these assays, we employed the human lung epithelial cell line A549 (ECACC No. 86012804), corresponding to type II epithelial cells from an adenocarcinoma cell line, which was obtained from the European Collection of Cell Cultures (ECACC). Cells were grown in Dulbecco's modified Eagle medium (DMEM) supplemented with 10% fetal bovine serum (FBS).

We also employed mouse alveolar macrophages transformed with SV40 (MH-S cell line), obtained from the European Cell Cultures Collection (ECACC No. 95090612). Cells were grown in RPMI 1640 medium plus 2mM glutamine (Invitrogen) + 0.05 mM 2-mercaptoethanol (Sigma Aldrich, USA) + 10% fetal bovine serum (Invitrogen).

Polymorphonuclear neutrophils (PMNs) were isolated from human blood samples taken from healthy volunteers. We used whole blood treated with anticoagulant EDTA. Briefly, a layer 5.0 ml of non-coagulated whole blood was placed over 5.0 ml of Polymorphprep in a 12 ml centrifuge tube. Centrifuge samples for 450 x g for 30 min. The polymorphonuclear fraction was then washed with Hanks' Balanced Salt Solution and centrifuged for 400 x g for 10 min. Finally, PMNs were resuspended in Dulbecco's Modified Eagle Medium (DMEM; Gibco), enumerated in hemacytometer and cell viability was determined using trypan blue [48]. PMNs were seeded into 24-well tissue culture plate and allowed to adhere for 20 min at 36°C with 5% of CO_2_. For inhibition of NADPH-oxidase, 10μM diphenylene iodinium (DPI; D2926, Sigma) was added to PMNs 20 min before infection.

For all assays, we used a ratio of 1:5 for *P*. *brasiliensis* yeasts: host cells. The interaction was kept at 36°C with 5% of CO_2,_ during 3h. After these interactions, SODs gene expression and colony forming units were determined to establish the percentage of viability [49,50].

### Construction of *P*. *brasiliensis PbSOD*1- and *PbSOD3-a*RNA yeast cells

DNA from Pb60855 was extracted from yeast cells cultures during exponential growth. We employed a Platinum high-fidelity Taq DNA polymerase (Invitrogen) to amplify aRNA oligonucleotides, designed on the sequences identified as PADG_07418 for *PbSOD1* gene [51] and PADG_02842 for *PbSOD3* gene. Gene sequences of the strain Pb03 were obtained from the *Paracoccidioides* genome database [30,31].

*P*. *brasiliensis* plasmid construction for aRNA and *Agrobacterium tumefaciens*-mediated transformation (ATMT) were performed as previously described [52]. Briefly, the amplified *PbSOD1* and *PbSOD3* aRNA oligonucleotides were independently inserted into the pCR35 plasmid under the control of the Calcium Binding Protein 1 (CBP-1) promoter region from *H*. *capsulatum* [53]. The pUR5750 plasmid was used as a parental binary vector to harbor this aRNA cassette within the transfer DNA (T-DNA). Constructed binary vectors were introduced into *A*. *tumefaciens* LBA1100 ultra competent cells by electroporation as described previously [54], and isolated by kanamycin selection (100 mg/ml).

In *P*. *brasiliensis* yeast cells (Pb60855), ATMT was done using *A*. *tumefaciens* cells harboring the desired binary vector, as described previously by Almeida et al. (2007) in order to obtain the knockdown strains. A 1:10 *P*. *brasiliensis/A*. *tumefaciens* ratio was employed during the 3 days period of co-culture at 28°C. Selection of *P*. *brasiliensis* transformants was performed in BHI solid media containing hygromycin B (Hyg; 200mg/ml) over a 15 days incubation period at 36°C. Randomly selected Hyg resistant transformants were tested for mitotic stability. This was determined by analyzing the stability of hygromicin B resistance. *PbSOD1*, *PbSOD3*-aRNA and PbEV strains were successively sub-cultured on BHI without hygromicin B (three consecutive rounds). Later on, we put them again under the selective pressure of the hygromicin B and analyzed them via amplification of the HPH cassette, verifying in this way the presence of the T-DNA. We analyzed more than one *PbSOD1*-aRNA and *PbSOD3*-aRNA strains, although results presented throughout the work refer to one selected aRNA strain per gene, and the same phenotypic characteristics were observed. *P*. *brasiliensis* yeast cells were also transformed with the empty vector pUR5750 (PbEV) as a control. In order to confirm the presence of the hygromycin B resistance cassette (HPH), PCR analysis was carried out to detect an HPH 1000-bp amplification product in PbEV, *PbSOD1*- and *PbSOD3*-aRNA strains.

### Vitality in *P*. *brasiliensis* yeast cells

Growth curves were performed in BHI broth (100 mL). We adjusted the inoculum to an OD of 0.4 for PbWT, PbEV and *PbSOD1*- *PbSOD3*-aRNA yeast cells. Then, cultures were incubated at 36°C and samples were collected at specific time points to determine growth curves by spectrophotometric analysis (OD_600_ nm; SmartSpec Plus (Bio-Rad, Hercules, CA).

Vitality was evaluated following the protocol reported by Hernandez et al. [55]. This corresponds to the ability of yeast cells to metabolize glucose upon late activation of a cell membrane proton pump and subsequent acidification of the medium due to H+ release [56]. Briefly, *P*. *brasiliensis* yeast cells were cultivated in BHI liquid medium and collected at exponential phase growth (72 h). Then, washed twice with sterile water pH 7.0. A 3 ml bottom was suspended in a final volume of 8 ml of water (pH 7.0) to obtain the yeast concentrated solution (YCS). Two milliliters of YCS were added to a beaker containing 38 ml of water pH 7.0. When the pH became stable (pH 5.5 to 6), 10 ml of 20% glucose were added. The pH was recorded every three min for 30 min, in order to evaluate changes in the pH of the medium. Also, as a negative control for the assay, PbWT yeast cells previously treated with 16 μg/ml of amphotericin B during 4h (Fungizone, Bristol-Myers Squibb Pharmaceuticals, England) were used [57]. The assays were performed in triplicates.

### Resistance of knockdown strains to oxidative stress-inducing agents

For the phenotypic analysis, we tested the sensitivity of the *PbSOD1* and *PbSOD3* knockdown strains to conditions inducing oxidative stress. The sensitivity to exogenous ROS of SOD mutants was analyzed using hydrogen peroxide, xanthine oxidase and menadione. Hydrogen peroxide sensitivity was tested using H_2_O_2_-saturated filter disks. 1×10^5^ *P*. *brasiliensis* yeast cells were spread on BHI plates. After inoculation, sterile filter disks were loaded with 20 μl of PBS1X as a control, and 0.5, 1, 2, 4 and 8 M of H_2_O_2_ (02194057, MP Biomedicals). Plates were incubated at 36°C with 5% CO_2_ and after eight days the area lacking *P*. *brasiliensis* growth was measured. For xanthine oxidase induced-oxidative stress, cells were incubated in 50 mM Tris pH 8, 100 μM hypoxanthine (H9636, Sigma) and 5 mU/ml xanthine oxidase (X4500, Sigma). Yeasts were incubated for 4 h at 36°C with shaking [20]. After this time, we plated serial dilutions of the experiment on Kurita’s medium in order to determine viable CFUs. Finally, menadione (M5625, Sigma) sensitiveness was evaluated in a 96-well plate. 1×10^4^ *P*. *brasiliensis* yeast cells were inoculated in each well, containing 0.5, 5, 10, 20, 40, 80 and 160 μM menadione. Plates were incubated at 36°C during eight days, after incubation time, the plate results were recorded.

### Mouse model of infection

Isogenic 6 to 8-week-old BALB/c male mice, obtained from the breeding colony of the Corporación para Investigaciones Biológicas (CIB), Medellín, Colombia, were used in assays and were kept with food and water *ad libitum* [58]. All animals were handled according to the national (Law 84 of 1989, Res No. 8430 of 1993) and international (Council of European Communities and Canadian Council of Animal Care, 1998) guidelines for animal research. The CIB research ethics committee approved the experimental protocols.

*P*. *brasiliensis* yeast cells were collected from exponentially growing batch cultures in BHI medium and counted using a hemacytometer. Animals (n = 5 per isolate) were infected with *P*. *brasiliensis* yeast by intranasal delivery of 1.5×10^6^ cells suspended in PBS buffer from PbWT, PbEV or *PbSOD1*- *PbSOD3*-aRNA strains. Mice were monitored daily for survival, weight loss and symptoms of disease. At 12 days post-infection, mice were euthanized and lung, liver and spleen tissues were homogenized in 2 mL PBS. Homogeneous suspensions were diluted (1:10, 1:100 and 1:1000) and 0.1 ml of each dilution was plated on Kurita’s medium [49] in order to determine the fungal burden in each organ. Plates were incubated at 36°C, 5% CO_2._ CFU counts were assessed ten days after cultivation. The data was transformed into Log10 CFU/g of tissue.

### Statistical analysis

Data are either the means or representative results of at least three similar repetitions, each performed in triplicate. Statistical analysis and comparisons were performed using paired Student’s *t* tests.

## Results

### *Paracoccidioides* genome encodes and expresses mRNA of six SOD isoforms

By means of sequence similarity and orthology analyses (BLAST and OrthoMCL, respectively), and using documented superoxide dismutase [59] sequences of reference fungal genomes, such as those of *Candida albicans*, *Aspergillus fumigatus* and *H*. *capsulatum*, we identified six conserved SOD homologs in the reference genomes of *Paracoccidioides* spp. (Pb18, Pb03 and Pb01) [30,31] as well as in Pb60855 (representing the PS3 lineage). Table 2 describes the Sod proteins encoded by the genome of *Paracoccidioides* spp. and the corresponding orthologs for each *PbSOD* in the Pb18, Pb03 and Pb01 genomes.

**Table 2:** SODs of *Paracoccidioides*.

Gene designation	Protein domain	Predicted Cellular localization*	*P. lutzii*	*P. brasiliensis*	
			Pb01	Pb03	Pb18
*SOD1*	Cu/Zn SOD	Cytosolic	PAAG_04164	PABG_03954	PADG_07418
*SOD2*	Fe/Mn SOD	Mitochondria	PAAG_02725	PABG_03204	PADG_01755
*SOD3*	Cu/Zn SOD	Extracellular	PAAG_02971	PABG_00431	PADG_02842
*SOD4*	Cu/Zn SOD, HMA	Cytosolic	PAAG_06501	PABG_02886	PADG_01400
*SOD5*	Fe/Mn SOD	Mitochondria	PAAG_02926	PABG_03387	PADG_01954
*SOD6*	Fe/Mn SOD	Cytosolic	PAAG_06363	PABG_11604	PADG_01263

*The predicted cellular localization was performed using TargetP 1.1 server [36]

**Fig 1:**
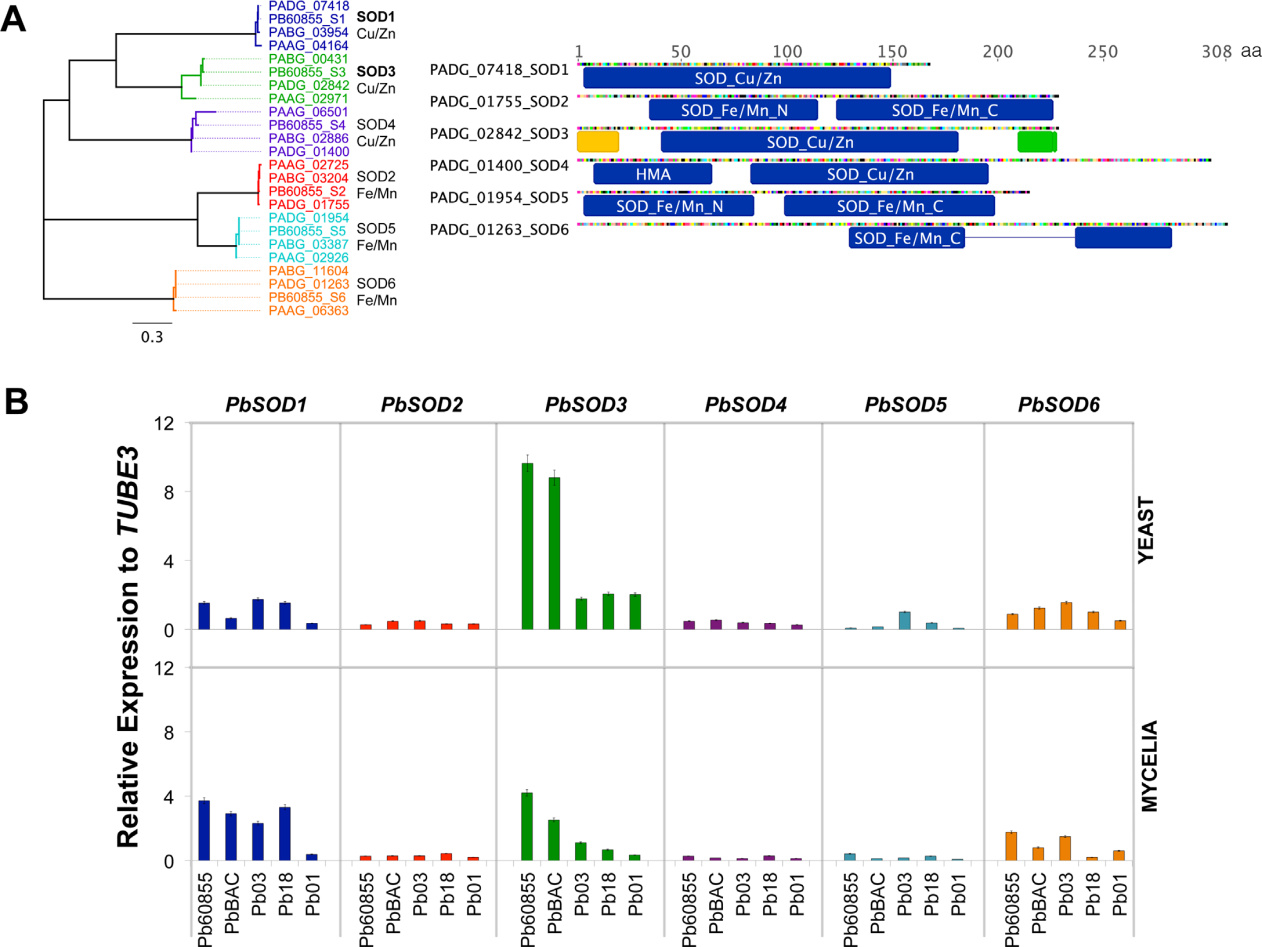
Identification, gene expression, and enzyme activity of superoxide dismutases encoded by *Paracoccidioides* spp. genome. **(A)** Phylogenetic tree, functional annotation and putative protein domains of identified SODs. **(B)** Expression profile of SOD isoforms in yeast and mycelia phases of the phylogenetic lineages of *Paracoccidioides* spp. One representative isolate of each one of the phylogenetic lineages of *Paracoccidioides* were chosen (Pb60855 and PbBAC correspond to PS3, Pb03 to PS2, Pb18 to S1 and Pb01 to *P*. *lutzii*.

This set of homologs encompasses the well-studied putative Sods, which include Sod1 (*PbSOD1*; PADG_07418; cytosolic), Sod2 (*PbSOD2*; PADG_01755; mitochondrial) and Sod3 (*PbSOD3*; PADG_02842; extracellular). Sequence analyses revealed three additional *SOD* homologs in *Paracoccidioides* (PADG_01400, PADG_01954, PADG_01263), which encoded putative Sods. These new genes were designated as *PbSOD4*, *PbSOD5* and *PbSOD6* respectively. *PbSOD4* shared sequence homology with the copper/zinc-dependent superoxide dismutases *PbSOD1* and *PbSOD3* genes. *PbSOD5* and *PbSOD6* shared sequence homology with the iron/manganese-dependent superoxide dismutase *PbSOD2* gene. Fig 1A shows the relationship between *SOD* homologs encoded by the genome of *Paracoccidioides* spp.

Protein family domain analysis using InterProScan showed that three of the Sod proteins (*PbSOD2*, *PbSOD5* and *PbSOD6*) had the Fe/Mn protein domains (PF00081: alpha N-terminal domain; PF02777: alpha/beta C-terminal domain). Unlike *PbSOD2* and *PbSOD5*, the *PbSOD6* protein sequence only had the Fe/Mn C-terminal domain divided into two intervals by 53 amino acids. Protein sequences of both *PbSOD2* and *PbSOD5* have both the Fe/Mn N-terminal and C-terminal domains conserving the Fe/Mn Sods putative structure (Fig 1A). In *A*. *fumigatus*, the *PbSOD6* homologous is a predicted mitochondrial protein that has an essential function to promote survival of the fungus, and as in the case of *Paracoccidioides*, it also lacks the N-terminus consensus sequence [27]. The three remaining identified Sod proteins (*PbSOD1*, *PbSOD3* and *PbSOD4*) have the Cu/Zn protein domain (PF00080; Cu/Zn superoxide dismutase; SODC). In addition to the Cu/Zn domain, the *PbSOD4* has a heavy metal-associated domain (HMA; PF00403) that is related to a metal iron transporter. In the case of *PbSOD3* we found a N-terminal signal peptide sequence (from 1 to 20 aa) using SignalP 4.0 [35], as long as an extracellular location pattern that was predicted using TargetP 1.1 [36]. There is also a C-terminal GPI-anchor/transmembrane signal (210–226 aa; Fig 1A) suggesting that this protein is associated with the cell surface. A previous study showed that *SOD3* of the dimorphic fungus *H*. *capsulatum* is part of the extracellular proteins produced by the pathogenic yeast phase and its localization allows *HcSOD3* to protect yeast cells specifically from exogenous superoxide [20]. Both results suggest that PbSod3p, as well as the HcSod3p are secreted proteins. Unlike *PbSOD3*, *PbSOD2* and *PbSOD5* have both a putative mitochondrial targeting signal as reported for other Mn/Fe *SOD2* [25,27]. Also, *PbSOD1*, *PbSOD4* and *PbSOD6* lack secreted-targeting sequences or mitochondrial targeting signals suggesting that these proteins are likely to be cytosolic. The identified *SOD* isoforms, their protein sequence domains and functional annotations are well conserved in the genomes of Pb18, Pb03, Pb60855 and Pb01, indicating that these proteins are part of the core genes of the *Paracoccidioides* genera.

We analyzed the gene expression level of each isoform in one representative isolate of the phylogenetic lineages of *Paracoccidioides* spp. (S1, PS2, PS3, and *P*. *lutzii*), in both mycelial and yeast cells during their exponential growth phase in batch culture. We observed that in PS3 isolates (Pb60855 and PbBAC) *PbSOD3* was the gene with the highest expression level in both phases, albeit higher in yeast cells (Fig 1B). *PbSOD1* was predominant in the mycelia phase in the S1, PS2 and *P*. *lutzii* strains. In Pb01, the expression level of all isoforms was lower when compared to that of *P*. *brasiliensis* isolates. Additionally, using a Sod zymogram we aimed at detecting if these isoforms were active and functional. First, we used a non-denaturing polyacrylamide gel (1D-PAGE) with crude extracts of *P*. *brasiliensis*, ATCC 60855: the cytoplasmic, the cell wall and extracellular fractions. The detection was performed following the method created by Beauchamp and Fridovich (1971). Despite all efforts, we only observed a minimum of two bands, which could be explained in part because the 1D-PAGE does not discriminate between isoforms with different molecular weight or isoelectric point (S2 Fig, top). To solve this, and in order to obtain a better resolution, we decided to carry out a 2D-PAGE (S2 Fig, bottom). Due to the characteristics of the technique, it was possible to observe three spots indicating the activity of a Sod isoform, without however specifically determining which achromatic zone corresponded to which isoform.

### *PbSOD3* is the most highly expressed isoform in the yeast phase and during the morphological changes in *P*. *brasiliensis*

*P*. *brasiliensis* cells respond to conditions such as i) variations in temperature (which induces morphological changes) and ii) continuous ROS production (a step required to carry out biological functions) with the induction of heat shock proteins [41,42,60] and enzymes that counteract ROS. As cell differentiation has been shown to be triggered by oxidative stress in different microbial eukaryotes, such as *C*. *albicans*, *A*. *nidulans* and *Neurospora crassa*, *inter alia* [61] and in order to better understand the possible role of SOD isoforms in *P*. *brasiliensis*, we analyzed the expression profile of one representative strain (*P*. *brasiliensis* ATCC 60855) in yeast and mycelia phases and during the morphological switch. *P*. *brasiliensis* conidia, mycelia and yeast cells were placed at either 36°C or 20°C in order to induce the transition (mycelia-yeast and conidia-yeast) and the germination (yeast-mycelia and conidia-mycelia) processes and a kinetic analysis of the expression of SOD isoforms was performed. In both the yeast and mycelia phases, *PbSOD1* and *PbSOD3* gene expression was higher during all evaluated time points, while *PbSOD2*, *PbSOD4* and *PbSOD5* were the less expressed genes (S3A Fig). *PbSOD3* was significantly expressed at higher levels in the yeast phase when compared to mycelia. On the other hand, *PbSOD1* was expressed at slightly higher levels in mycelia when compared to the yeast phase (S3A Fig). Regarding the morphological shift, *PbSOD1* and *PbSOD3* showed increased expression during the Y-M and M-Y morphological shifts (S3B Fig). *PbSOD4* and *PbSOD5* were the less expressed genes during all evaluated time points and conditions. During the Y-M germination and M-Y transition, *PbSOD1* and *PbSOD3* were the most expressed isoforms (S3B Fig). Overall, expression levels in experiments involving morphological shifts from conidia were low for all *SOD* isoforms with the exception of during the C-M germination in which *PbSOD2* gene expression increased throughout time, peaking at 96 hours, and the initial hours (3 and 12) of the C-Y transition in which *PbSOD6* increased its expression.

### *PbSOD3* is highly expressed during interaction with human host cells

**Fig 2:**
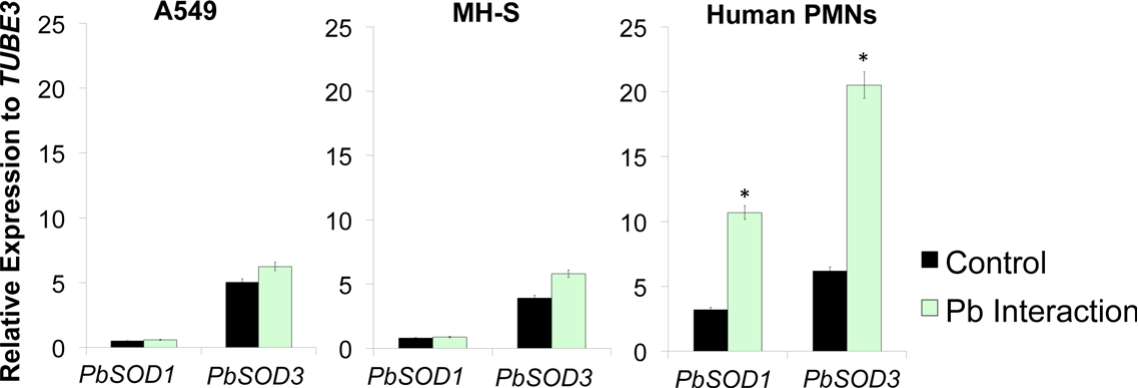
Expression of *PbSOD* isoforms during A549, MH-S cell line, and Human PMNs interactions. *PbSOD* isoforms gene expression in PbWT60855 yeast cells, co-cultivated in the presence and in the absence of pneumocytes (cell line A549), Macrophages (cell line MH-S) and Human PMNs. Gene expression levels were obtained by RT-qPCR assay and normalized with the housekeeping gene β-tubulin. Results are the mean of three individual experiments. Asterisks denotes *P* ≤0.05 compared to PbWT.

We determined the expression of SOD isoforms during the interaction with pneumocytes (cell line A549), since these cells would be the first barrier faced by *Paracoccidioides* cells when trying to adhere to the host lung tissue and thus establishing the infection [55,62]. We found that the interaction of *P*. *brasiliensis* yeast cells with A549 cells induced a slight increase in *PbSOD3* gene expression (Fig 2). The gene expression of the other isoforms remained unaltered when compared to the unchallenged control. Subsequently, we analyzed gene expression of SOD isoforms during the interaction with phagocytic cells [alveolar macrophages (cell line MH-S) and human PMNs] since after the initial establishment of the infectious process, *Paracoccidioides* cells must counteract the phagocytic arsenal elicited by macrophages and PMNs in order to establish itself within the host. During the interaction of *P*. *brasiliensis* yeast cells with human PMNs we observed an increase of *PbSOD1* gene expression of almost 2-fold, when compared to the unchallenged control. However, upon interaction with human PMNs, *PbSOD3* gene expression was much higher (almost 4-fold) when compared to unchallenged yeast cells (Fig 2). Gene expression of the other isoforms remained unaltered.

### Silencing of *PbSOD1* and *PbSOD3* genes has no detrimental effect on *P*. *brasiliensis* yeast cells growth rate

**Fig 3:**
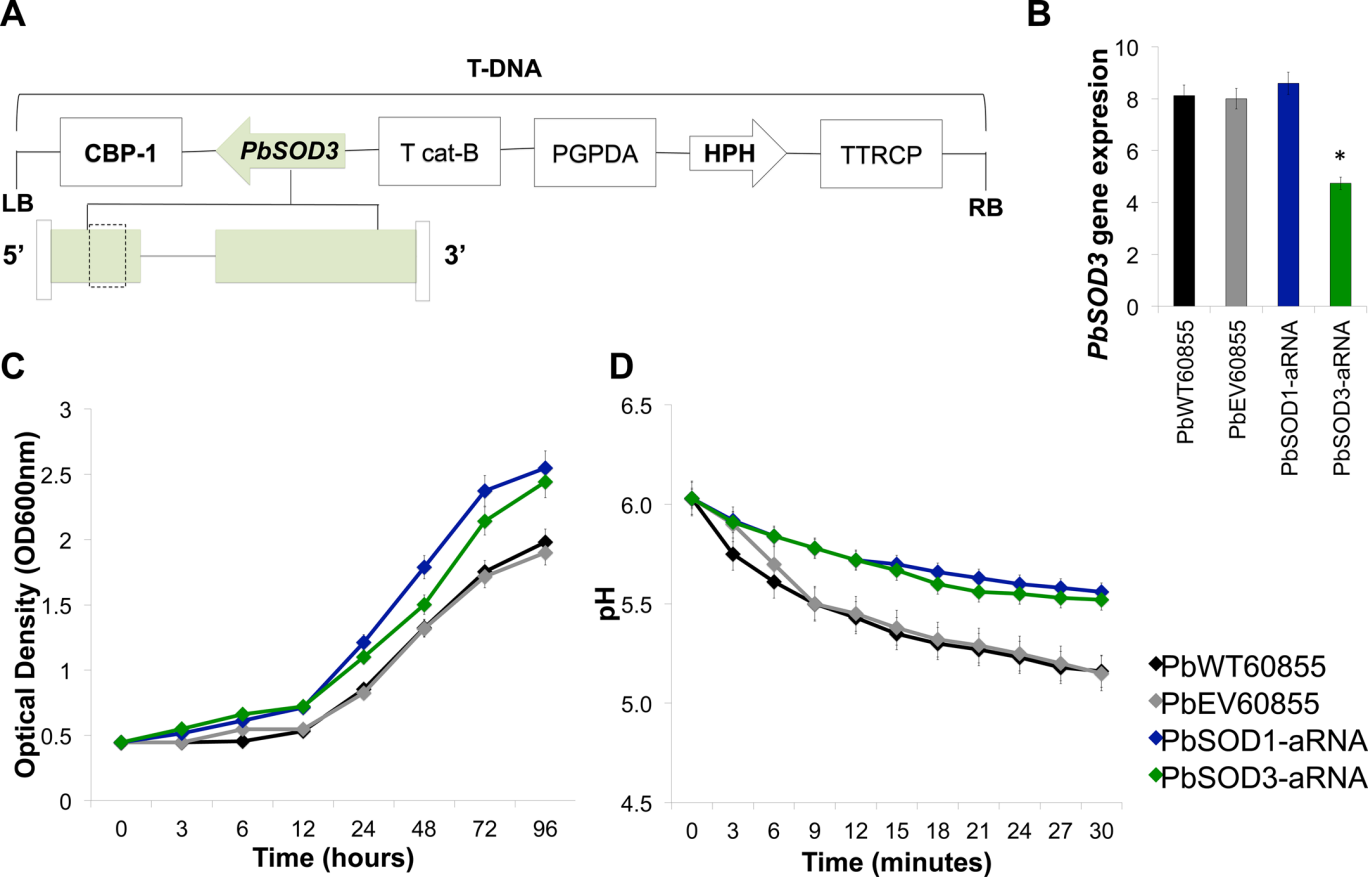
Silencing of *SOD1* and *SOD3* in *P*. *brasiliensis*. **(A)** Transfer DNA (T-DNA) inserted into the genome of *P*. *brasiliensis* yeast cells via ATMT in order to silence *PbSOD3* gene/protein. The antisense oligonucleotide was directed to exon 1 (dashed box), with a length of 104 bp. This AS oligonucleotide was placed under control of the calcium binding protein (*CBP*-1), the terminator (*CAT*-B) and harbored hygromycin B phosphotransferase (*HPH*) under control of glyceraldehyde 3-phosphate of *Aspergillus nidulans* (*PGPDA*) and with the terminator (*TTRCP*). **(B)** Gene expression levels of *PbSOD3* obtained by RT-qPCR assay. The measurement was normalized with the housekeeping gene β-tubulin in PbWT, PbEV and Pb*SOD3*-aRNA yeast cells grown at exponential phase. Mitotic stability was confirmed by sub-culturing *P*. *brasiliensis PbSOD3*-aRNA yeast cells, checking for low expression levels in this isolate after successive sub-cultures. Results are the mean of three individual experiments. Asterisk represent significant differences compared to PbWT and PbEV as determined by Student’s *t* test. **(C)** Growth curve in PbWT60855, PbEV60855, *PbSOD1*-aRNA and *PbSOD3*-aRNA. Yeast cells were grown in BHI liquid medium at 36°C, OD600 nm was determined along each time point. **(D)** Vitality in PbWT60855, PbEV60855, *PbSOD1*-aRNA and *PbSOD3*-aRNA. The pH in yeast suspensions was monitored at three minutes intervals for 30 minutes.

To further analyze the function of *SOD1* and *SOD3* in *P*. *brasiliensis*, we used an antisense-RNA (aRNA) knockdown strain for *SOD1* (*PbSOD1*-aRNA), which was previously constructed [51] (S4 Fig) and a *PbSOD3*-aRNA knockdown strain constructed in this study using ATMT. Briefly, one region of the gene was selected in order to design two different aRNA oligonucleotides and generate knockdown strains. Both of them were directed at exon 1 (107-bp for *PbSOD3AS1* and 104-bp for *PbSOD3AS2*; Fig 3A). We selected a mitotically stable isolate with the highest decrease in *PbSOD3* gene expression, which ranged between 50 to 70% (strain *PbSOD3*-aRNA; Fig 3B). The insertion of the transfer DNA (tDNA) into *P*. *brasiliensis’* genome was confirmed via amplification of the HPH cassette (S5 Fig).

We initially analyzed possible alterations during batch culture of the knockdown strains (*PbSOD1*-aRNA and *PbSOD3*-aRNA). No major changes were detected during growth curve analysis of either knockdown strain when compared to PbWT or PbEV. However, a decreased capacity to metabolize glucose in both *PbSOD1*-aRNA and *PbSOD3*-aRNA strains as compared to PbWT and PbEV yeast cells was observed, detected as an increase in the pH, (Fig 3C), indicating reduced yeast cell vitality of the knockdown strains.

### *PbSOD1*- and *PbSOD3*-aRNA strains are less resistant to oxidative agents, but only *PbSOD3*-aRNA shows increased sensitivity to xanthine oxidase-induced oxidative stress

**Fig 4:**
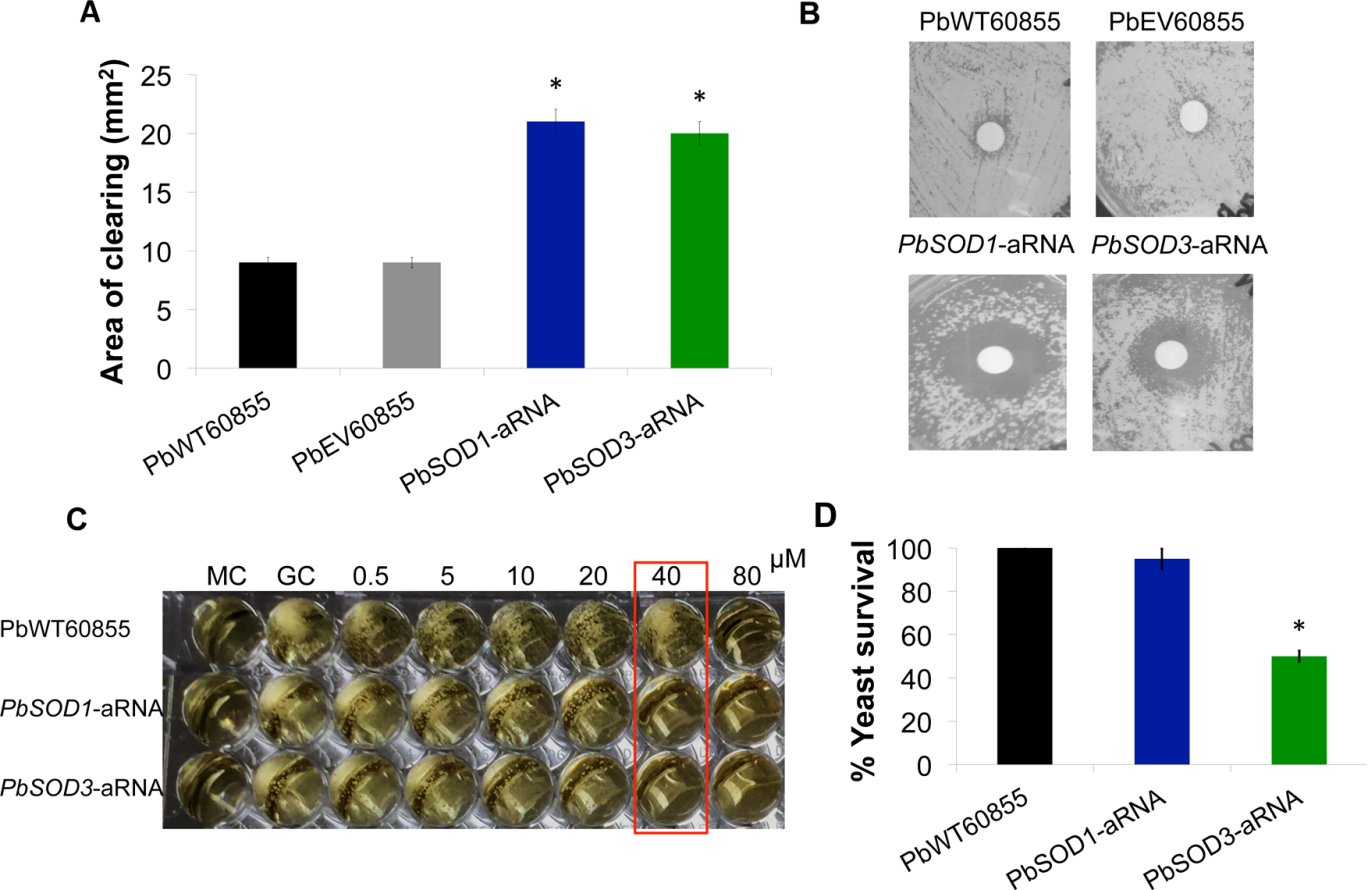
Exposure of SOD knockdown strains to ROS-inducing agents. **(A)** Quantification of the sensitivity to H_2_O_2_ in *P*. *brasiliensis* yeast cells. The zone of inhibition was determined as the area lacking yeast cell growth. Data presented are the average for three replicate tests, with error bars representing standard deviations. Asterisks represent significant differences from the wild-type strain as determined by Student’s *t* test. **(B)** Images of *P*. *brasiliensis* growth around H_2_O_2_ saturated disks. Saturated filter disks containing 1M H_2_O_2_**. (C)** Sensitivity to menadione of *PbSOD1* and *PbSOD3* mutants. We used a 96-well plate in which a total of 1X10^4^ yeast cells of each isolate were inoculated on BHI containing increased amounts of menadione (0.5, 5, 10, 20, 40, 80 and 160 μM). The plates were incubated at 36°C, with 5% of CO_2_ for 8 days. The red box indicates the concentration at which no growth in *PbSOD1*-aRNA and *PbSOD3*-aRNA occurred, contrary wise to PbWT60855 in which we observed growth. **(D)** Survival of *P*. *brasiliensis* yeast cells following challenge with xanthine oxidase. Yeast cells were incubated for 4h at 36°C in the presence of hypoxanthine 100 μM and xanthine oxidase 5 mU/ml in order to generate superoxide anions. After incubation time passed, viable CFUs were determined. Results are the mean of three individual experiments. Asterisk denotes *P* ≤0.05 compared to PbWT and PbEV as determined by Student’s *t* test.

To further evaluate the role of PbSod1p and PbSod3p, we analyzed the effect of oxidative stress-inducing agents (hydrogen peroxide, xanthine oxidase and menadione) on fungal growth of the knockdown strains. *PbSOD1*-aRNA and *PbSOD3*-aRNA yeast cells were exposed to hydrogen peroxide using a disk-diffusion assay with increasing concentrations of the compound (0.5, 1, 2, 4 and 8 M). Knockdown strains showed a higher sensitivity to H_2_O_2_ with the largest clearing areas (Fig 4A and 4B), although no major differences were detected between *PbSOD1*-aRNA and *PbSOD3*-aRNA.

Taking into account that the main substrate for *SOD1* and *SOD3* is the superoxide anion [63], we also induced oxidative stress using xanthine oxidase and menadione, compounds known to be superoxide-generating agents. Menadione generates ROS through redox cycling [64], while xanthine oxidase generates ROS catalyzing the oxidation of substrates, such as purines (xanthine and hypoxanthine), and a variety of electron acceptors such as O_2_ and NAD+, which react with the enzyme [65]. Both *PbSOD1*-aRNA and *PbSOD3*-aRNA yeast cells were less resistant to menadione when compared to the control (Fig 4C). Furthermore, we used xanthine oxidase 5 mU/ml and hypoxanthine 100 μM to generate superoxide *in vitro*. Interestingly, *PbSOD3-*aRNA yeast cells were more sensitive to this oxidative stress-inducing agent than the *PbSOD1*-aRNA and PbWT strains (Fig 4D).

### *PbSOD3* is required for virulence of *P*. *brasiliensis* yeast cells

**Fig 5:**
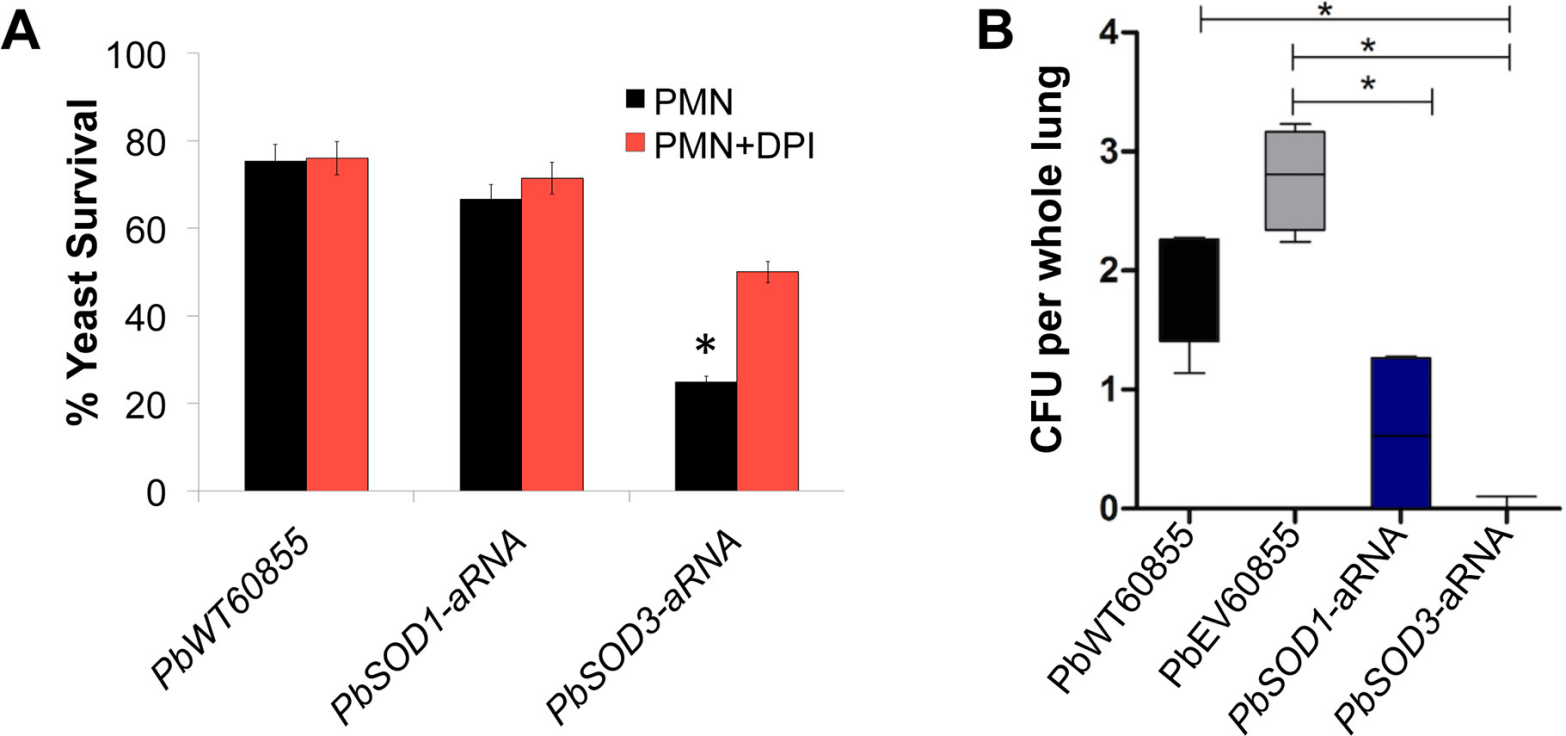
Effect of *SOD1* and *SOD3* decreased expression in an *in vitro* and *in vivo* model. **(A)** *P*. *brasiliensis* yeast cells were incubated in the presence of human PMNs for 3 h, at 36°C, with 5% of CO_2_. After this, survival of yeast cells was determined by enumeration of viable CFUs (black bar). Later on, human PMNs were treated with DPI (red bar) in order to inhibit NADPH-oxidase. Subsequently, yeast survival was determined during the interaction of *P*. *brasiliensis* with human PMNs treated with DPI. This lead to an increase in the survival of *PbSOD3*-aRNA isolate, that correlated with the inhibition of the ROS-mediated killing. **(B)** Virulence of *PbSOD1*- and *PbSOD3*-aRNA isolates in an experimental murine model of infection. CFUs recovered from lungs of mice intranasally inoculated with 1.5X10^6^ yeast cells obtained from PbWT, PbEV, and *PbSOD1*- and *PbSOD3*-aRNA strains. Mice were euthanized at 12 days post-infection, organs were harvested and the fungal burden in lung tissue was determined. No significant differences between the PbWT and PbEV strains were found. Asterisks indicate significant differences from animals infected with PbWT and PbEV as compared to *PbSOD1*- and *PbSOD3*-aRNA strains determined by Student’s *t* test.

To test the involvement of Sod1p/Sod3p in phagocyte-defense, human PMNs were incubated with both knockdown and wild-type yeast cells. As the fungicidal effect of PMNs on fungal cells depends mostly on the production of ROS, we also investigated the effect of the suppression of NADPH oxidase, required for the activation of the oxidative burst and subsequent generation of ROS in PMNs. In order to inhibit NADPH oxidase we treated human PMNs with DPI prior to challenging with yeast cells. PbEV had the same behavior as PbWT yeast cells. It is important to note that *PbSOD3*-aRNA yeast cells were more susceptible to PMN’s fungicidal ability than *PbSOD1*-aRNA. Furthermore, treatment with DPI considerably reduced PMN’s ability to kill *PbSOD3*-aRNA strain (from 25 to 50%; Fig 5A). Evaluation of *PbSOD1* and *PbSOD3* gene expression in *P*. *brasiliensis* yeast cells challenged with human PMNs was also performed. As shown previously, the interaction with PMNs led to increased expression of *PbSOD1* and *PbSOD3*, both in the presence–to a greater extent–and absence of DPI (S6 Fig). *PbSOD1*-aRNA and *PbSOD3*-aRNA strains showed a similar behavior but with reduced expression levels most likely due to knockdown of the gene expression.

To further analyze the involvement of Sod1p and Sod3p in *P*. *brasiliensis* virulence, mice were infected intranasally with 1.5×10^6^ *PbSOD1*-aRNA or *PbSOD3*-aRNA yeast cells. CFU in mouse lungs, kidneys and spleen were determined on the twelfth day after infection, which matches with the onset of the cell-mediated immune response [20]. We found active infection in lungs of PbWT, and PbEV, while in the *PbSOD1*-aRNA strain there was a significantly lower fungal burden. Moreover, regarding the *PbSOD3*-aRNA strain, no CFUs were detected (Fig 5B). CFUs were recovered from the liver of mice infected with PbWT and PbEV strains, but not from tissues of mice infected with the *PbSOD1*-aRNA or *PbSOD3*-aRNA strain. CFUs were not recovered from the spleen in any of the infected mice, irrespective of the strain used.

## Discussion

This is the first study on the SOD family, the largest antioxidant gene family identified so far in the Ajellomycetaceae, specifically in the *Paracoccidioides* genus. We report the existence of six SOD isoforms encoded by the *Paracoccidioides’* genome. Despite our efforts and due to technical difficulties, we were only able to detect biochemical evidence for the presence of three isoforms (S2 Fig). Additionally, we could not identify to which isoform corresponded each achromatic zone, due to the as-of-date lack of methods to generate knockout strains in *P*. *brasiliensis*. Through quantitative RT-qPCR, we determined the profile expression of each isoform in different conditions. *PbSOD2*, *PbSOD4*, *PbSOD5* and *PbSOD6* did not show differential expression in any phase (yeast or mycelia), morphological shift or during interaction with host cells, while *PbSOD1* and *PbSOD3* were differentially expressed depending on the growth conditions and stimuli.

*PbSOD3* gene expression was predominant in the yeast phase of the PS3 isolates, unlike S1, PS2 and *P*. *lutzii* isolates, where there was no such a differential expression of this isoform (Fig 1B). On the other hand, *PbSOD1* and *PbSOD3* were similarly expressed during the yeast phase, while in mycelia *PbSOD1* expression was slightly higher for S1, PS2 and *P*. *lutzii* isolates. Importantly, differences in the metabolic profile among the members of the *Paracoccidioides* genus have been detected [66], similar to as shown in the *Histoplasma* genus [67], which could underlie distinct capabilities to survive phagocytic microbicidal attack set by the host. In the case of *H*. *capsulatum*, strain G217B is equipped with an improved antioxidant defense system while in G186A the evasion of phagocyte detection is critical for virulence [67]. Regarding *Paracoccidioides* spp., a recent study demonstrated that *P*. *lutzii* has a more active anaerobic metabolism than *P*. *brasiliensis* [66]; which correlates with the lower expression level of the studied *SOD* isoforms in *P*. *lutzii*. Since these cells have a reduced oxygen consumption they would most likely not have to synthesize such an elevated level of proteins related to oxidative stress defense as *P*. *brasiliensis*, particularly during host-pathogen interaction at the onset of the infection. Additionally, we should considerer that although both species and isolates from all phylogenetic lineages can infect humans and produce Paracoccidioidomycosis (PCM), they can also vary in virulence and induce different immune responses [68]. This issue is critical and needs to be elucidated in order to better understand the pathobiology of PCM and how it may relate to the different species and lineages of *Paracoccidioides* spp.

Regarding to the morphological switch we detected that during the M-Y transition and in the yeast phase, *PbSOD3* was expressed at higher levels (S3A and S3B Fig). This suggests that the gene may display a phase-dependent expression and possibly be required for the defense against ROS produced endogenously (as a consequence of the high temperature and increased metabolism) and exogenously (as a consequence of the infectious process). Importantly, *Paracoccidioides*, *H*. *capsulatum* and *Blastomyces SOD3* have a conserved predicted signal peptide and a glycophosphatidyl inositol (GPI) anchor (Fig 1A) [15, 28], which could allow the cell to excrete the protein and to associate with the cell surface, suggesting its role as an extracellular Sod. Overall, these data might suggest a role in protecting *Paracoccidioides* yeast cells against the conditions faced once within the human host (*e*.*g*. increase in temperature) and also when defending against phagocytes-induced ROS. To test our hypothesis, we used host cells in order to investigate which SODs were involved during the initial host-pathogen interactions. A549 and MH-S cells did not significantly trigger SOD induction, but human PMNs induced the expression of only two of the six SODs, *PbSOD1* -to a lesser extent- and *PbSOD3*, the latter at higher levels (Fig 2). *P*. *brasiliensis* yeast cells posses some mechanisms in order to evade MH-S cells, including the production of melanin (*in vivo* and *in vitro)* [69], which can interfere directly with the binding of the fungi to phagocytic cells [70]. In addition to the melanin, *P*. *brasiliensis* also produce an extracellular antigen (Gp43) that is able to interact with host cells, inhibiting both the phagocytosis and the releasing of nitric oxide (NO) by macrophages [71,72]. Therefore, macrophages are not able to produce ROS in response to *P*. *brasiliensis* infection [71]. This could explain the low induction of SOD isoforms during interaction with both, A549 (pneumocytes) and MH-S cell lines.

Studies in *P*. *brasiliensis* have focused on studying the role of neutrophils during the initial stages of the infection and during the development of PCM. Neutrophils play a relevant role in host defense and the resistance mechanisms against PCM, exerting an immunoregulatory role in antibodies and cytokine secretion during the course of the disease [73]. These cells are most frequently associated with extracellular killing mechanisms that involve the release of large amounts of ROS and granule components in the extracellular medium [74,75]. This was demonstrated in vitro for *P*. *brasiliensis*. Human PMNs ingest yeast through phagocytosis but when these are too large they form an extracellular vacuole in order to kill both ingested and extracellular cells [76]. These facts together with the presence of high activities of NADPH oxidase during the phagocytosis of *P*. *brasiliensis* by neutrophils [76] are in line with our results regarding the induction of antioxidant enzymes SOD1 and SOD3 during PMN-*P*. *brasiliensis* interaction.

Accordingly, we generated the knockdown strains for either gene and observed that both knockdown strains had reduced vitalities during batch culture growth (Fig 3D). The high concentration of glucose used during the vitality test accelerates glycolysis in the cells, leading to ATP synthesis (necessary for metabolizing glucose) and the consequent production of ROS [77]. We attributed these low vitalities to the fact that PbWT could more readily counteract ROS produced as a consequence of the glucose metabolism, contrarily to the aRNA strains. The decreased cell vitality could be indicating metabolic alterations in *PbSOD1*-aRNA and *PbSOD3*-aRNA yeast cells, that could also affect the performance of the pathogen either during batch culture growth or during the host-pathogen interactions.

Furthermore, when we challenged knockdown strains with H_2_O_2_, menadione and xanthine oxidase, we found that both knockdown strains were both similarly more susceptible to H_2_O_2_- and menadione-induced oxidative stress than the wild-type strain (Fig 4A, 4B and 4C). Although H_2_O_2_ is not a Sod substrate, it has been previously established that treatment with this compound in *Saccharomyces cerevisiae* cells induces the transcription of SODs [11,47], implying in some way an indirect involvement of these enzymes in defense against H_2_O_2_. We also measured the gene expression of SODs after the interaction with this compound, and found that both *PbSOD1* and *PbSOD3* genes were induced (S7 Fig). Additionally, H_2_O_2_ is able to inactivate SOD activity, in a concentration and time-dependent fashion [78]. It has also been proven that cytosolic, extracellular and mitochondrial SODs have peroxidase properties [79,80], which would enable the enzyme to directly interact with its product H_2_O_2_. In order to prove this in *P*. *brasiliensis* cells, we determined the ability of the wild-type strain and the aRNA strains to eliminate H_2_O_2_ *in vitro*. In agreement with the evidence that suggests that *SOD3* acts at an extracellular level, *PbSOD3*-aRNA strain had a decreased ability to destroy H_2_O_2_ in the extracellular and cell wall fractions. *PbSOD1*-aRNA strain had a decreased ability to degrade H_2_O_2_ in the intracellular fraction. PbWT and PbEV strains had similar behaviors in all evaluated cellular extracts, with a higher capability to destroy this compound when compared to aRNA strains (S8 Fig). These data may further support the involvement of *PbSOD1* and *PbSOD3* in defending *P*. *brasiliensis* cells not only against superoxide anions but also against peroxide-induced oxidative stress.

Menadione induces endogenous oxidative stress generating superoxide anions through redox cycling. Notably, we found that *PbSOD1*-aRNA and oddly *PbSOD3*-aRNA were similarly susceptible to this compound. In this respect, it was demonstrated for *S*. *cerevisiae* cells that superoxide anions generated by menadione also acts and are formed at the outside of the cell, and consequently, the addition of SOD into the incubation buffer (acting as an extracellular enzyme) protected cells from cytotoxic effects of the compound [81]. This fact attests to the relevance of the extracellular enzyme in defending the cell against superoxide anions generated by menadione. As the anions generated extracellularly do not readily diffuse across the plasma membrane, the toxic effects might also occur in the outside of the cell; which is aligned with our finding of *PbSOD3*-aRNA strain being as sensitive as *PbSOD1*-aRNA to menadione-induced oxidative stress, supporting that both, extracellular and intracellular enzymes are required for defending yeast cells against menadione-induced oxidative stress. Accordingly, we observed a higher susceptibility of *PbSOD3*-aRNA yeast cells to xanthine oxidase-induced oxidative stress than in *PbSOD1*-aRNA (Fig 4D). As in the menadione-induced oxidative stress, the superoxide anions generated by xanthine oxidase and the lack of diffusion of them across membranes may maintain higher levels of ROS outside of the fungal cell, and consequently *Paracoccidioides* yeast cells should require SOD enzymes capable of acting in an extracellular level in order to counteract the detrimental effects of this ROS. This result may be related to the fact that Sod3p most likely counteracts exogenous superoxide and that in the *PbSOD3*-aRNA strain this activity is partially lost, presenting increased susceptibility to xanthine oxidase-induced oxidative stress. Moreover, despite the fact that *Paracoccidioides* cells have and express other Sods (Sod1, Sod2, Sod4, Sod5, and Sod6) it is possible that Sod3 is potent enough to counteract exogenously-produced ROS under the studied conditions. In summary, defense against endogenous-produced ROS may depend on intracellular Sods (mostly Sod1p, but also could be involved Sod2p, Sod4p, Sod5p and Sod6p), but defense against extracellular-produced ROS (produced during host-pathogen interactions) might rely mostly on Sod3p [20,25,27]. Nonetheless, this needs to be further elucidated.

We employed yeast co-cultured with human PMN and a murine model of infection to study the involvement of *PbSOD1* and *PbSOD3* isoforms in *Paracoccidioides* virulence. We observed that *P*. *brasiliensis* cells are highly resistant to the action of phagocytes and that the *PbSOD3*-aRNA strain was significantly more susceptible to the action of PMNs than *PbSOD1*-aRNA and the control strains (PbWT and PbEV; Fig 5A). Later on, we confirmed that the increased susceptibility of *PbSOD3*-aRNA strain to PMNs accounted for the oxidative mechanism and not for the polypeptide antibiotics delivered from lysosomal granules [82] using DPI to specifically inhibit NADPH-oxidase. These results showed a reduction in the killing by PMNs in *PbSOD3*-aRNA, likely related to the inhibition of the oxidative burst in PMNs and the reduction in the expression of the extracellular Sod (*PbSOD3*; Figs 5A and S6). Furthermore, results using a mouse model of infection indicated that *PbSOD3* seems to be more relevant in the establishment and development of the PCM, since yeast cells with decreased *PbSOD3* gene expression were unable to infect lung tissues. Meanwhile, the *PbSOD1*-aRNA strain was able to infect the lungs, but unable to disseminate to other organs (Fig 5B). In agreement with this, *H*. *capsulatum* knockout cells for *SOD3* showed that this gene is required for full virulence *in vivo*, while the absence of the *SOD1* gene did not impair lung infection [20]. In addition, in *Blastomyces* it has been also shown the up-regulation of *SOD3* during the interaction with macrophages and in a mouse model of infection [83]. Thus, our results may suggest that Sod3p may play an important role in *P*. *brasiliensis* virulence, either the establishment of the infectious process or dissemination, while Sod1p, although not essential for establishing the respiratory disease, still might be required for fungal dissemination.

Based on our results, we postulate that *P*. *brasiliensis* actively responds to the radicals generated endogenously during metabolism and counteracts the oxidative burst of immune cells by inducing the expression of SOD isoforms. In the former case, they could specifically induce *PbSOD3* gene expression endowed with an extracellular activity and therefore might be considered an essential gene during the events underlying the host-pathogen interaction.

## Supporting Information

S1 Table qPCR primers used in this study. (DOCX)

S1 Fig Housekeeping genes employed for expression experiments. **(A)** *PbTUBE3* gene expression in PbWT60855 cells undergoing morphological switches and during batch culture growth. Gene expression levels were obtained by RT-qPCR assay in order to discard any variation in *PbTUBE3* gene expression, confirming in this way that this gene can be used as normalizer under the experimental conditions carried out in this work. The measurement was normalized using as housekeeping gene *PbTEF3* in cells undergoing transition (C-Y, M-Y) and germination (C-M, Y-M) processes, and during a batch culture growth. **(B)** Expression of SOD isoforms during transition from M-Y, and during germination from Y-M. Normalization was performed using *TEF3*.
(TIF)

S2 Fig Active Sod isozymes detected in crude extracts of Pb60855. **Top:** Sod activity in a non-denaturing polyacrylamide gel (1D-PAGE; 150 μg of crude protein extract; *C* = cytoplasmic fraction, *CW* = cell wall fraction). In the 1D-PAGE at least two bands can be observed, indicating the Sod activity. In order to obtain a better gel resolution and visualize the six isoforms, a 2D-PAGE was carried out. **Bottom:** Sod activity in a non-denaturing 2D-PAGE (150 μg of crude protein extract). Illustration shows three spots indicating the activity of Sod isoforms. We were unable to predict to which isoform corresponded every band or spot, but it is clear that they corresponded to the Sods.
(TIF)

S3 Fig Kinetic expression of SOD isoforms in *P*. *brasiliensis* yeast cells. **(A)** Kinetic expressions during batch culture growth, *PbSODs* gene expression was evaluated in Pb60855 yeast and mycelia forms. **(B)** Expression of SOD isoforms during transition from C-Y and from M-Y, and during germination from C-M and from Y-M. Higher expression levels of *PbSOD3* during all evaluated events can be observed. During the transition from C-Y and germination from C-M, the expression level of all isoforms was quite similar. On the other hand, levels of *SOD1* and *SOD3* became more evident during transition from the M-Y and germination from the Y-M.
(TIF)

S4 Fig *Paracoccidioides* S*OD1* gene expression in PbWT60855, PbEV60855 and *PbSOD1*-aRNA yeast cells during batch culture growth. Gene expression levels of *PbSOD1* were obtained by RT-qPCR assay. The measurement was normalized with the housekeeping gene β-tubulin in PbWT, PbEV and Pb*SOD1*-aRNA yeast cells grown at exponential phase. Results are the mean of three individual experiments. Asterisks denotes *P* ≤0.05 compared to PbWT.
(TIF)

S5 Fig Validation via PCR of the presence and integration of the Transfer DNA (T-DNA) into the genome of *P*. *brasiliensis*. Genomic DNA from the PbWT60855, PbEV60855 and the knockdown strains *PbSOD1*-aRNA and *PbSOD3*-aRNA were tested by PCR using specific primers for the tubulin gene (*TUB)* and for the hygromycin B resistance gene (HPH).
(TIF)

S6 Fig *SOD1* and *SOD3* gene expression levels in *PbSOD* knockdown strains during interaction with human PMNs. *PbSOD1* and *PbSOD3* gene expression were determined through RT-qPCR assay and normalized with the housekeeping gene β-tubulin. Results are the mean of three individual experiments. Asterisks denotes *P* ≤0.05 compared to PbWT.
(TIF)

S7 Fig *SOD1* and *SOD3* gene expression levels in PbWT strain after incubation in the presence of hydrogen peroxide (30mM) and menadione (1mM). *PbSOD1* and *PbSOD3* gene expression were determined through RT-qPCR assay and normalized with the housekeeping gene β-tubulin. Results are the mean of three individual experiments.
(TIF)

S8 Fig Determination of the ability of *P*. *brasiliensis* yeast cells to degrade H_2_O_2._ Extracellular, cell wall and cytoplasmic fractions of PbWT, PbEV, *PbSOD1*-aRNA and *PbSOD3*-aRNA strains were collected. Relative H_2_O_2_ destruction was measured as the decrease in the absorbance at 240 nm. Results are the mean of three individual experiments.
(TIF)
