# Supplementary material for: Identification and Analysis of the Role of Superoxide Dismutases Isoforms in the Pathogenesis of Paracoccidioides spp
Source: PLoS Negl Trop Dis. 2016 Mar 10;10(3):e0004481. doi: 10.1371/journal.pntd.0004481 (PMC4786090; doi:10.1371/journal.pntd.0004481)
Supplement: S1 Table — (DOCX) [file pntd.0004481.s001.docx]

**S1 Table.** qPCR primers used in this study.

| **Gene** | **Exon** | **Sequence 5´🡪 3´** |
| --- | --- | --- |
| ***SOD1*** | 2 | F: 5´TGTCAAGGGCACTGTAGTCTTC 3´ |
|  |  | R: 5´TTTGGGTCGTTGCCAGAAAG 3´ |
| ***SOD2*** | 2 | F: 5´TCCACCACAAAAAGCACCAC 3´ |
|  |  | R: 5´AGCCACCTGGGATTTGATATCG 3´ |
| ***P. brasiliensis* *SOD3*** | 1 | F: 5´ATACCACTGTTCGCGGAGTT 3´ |
|  |  | R: 5´GGCATGGTACATCACTCCAA 3´ |
| ***P. lutzii* *SOD3*** | 1 | F: 5´ATTCGAGCCGTATCAACTGC 3´ |
|  |  | R: 5´AAGGCACCAACACGATTTGC 3´ |
| ***SOD4*** | 1 | F: 5´GTTGCGGTATGGGAGTTGATTG 3´ |
|  |  | R: 5´ATAACTCCCACCAGCGTGTTC 3´ |
| ***SOD5*** | 2 | F: 5´TCCGTCGCAAATTCAACGAG 3´ |
|  |  | R: 5´TGATCCTGCGTCGTGGTAATAG 3´ |
| ***SOD6*** | 4 | F: 5´TATGCTGATGCCCACTACCG 3´ |
|  |  | R: 5´GTTCTCTCCTCCGGTGATGC 3´ |
| **β-Tubuline** | 3 | F: 5´TCCCTTTGGCGAACTCTTTC 3´ |
|  |  | R: 5´TTGTTTCCAGCACCAGACTG 3´ |
