# Supplementary figures and images for: Identification and Analysis of the Role of Superoxide Dismutases Isoforms in the Pathogenesis of Paracoccidioides spp
Source: PLoS Negl Trop Dis. 2016 Mar 10;10(3):e0004481. doi: 10.1371/journal.pntd.0004481 (PMC4786090; doi:10.1371/journal.pntd.0004481)

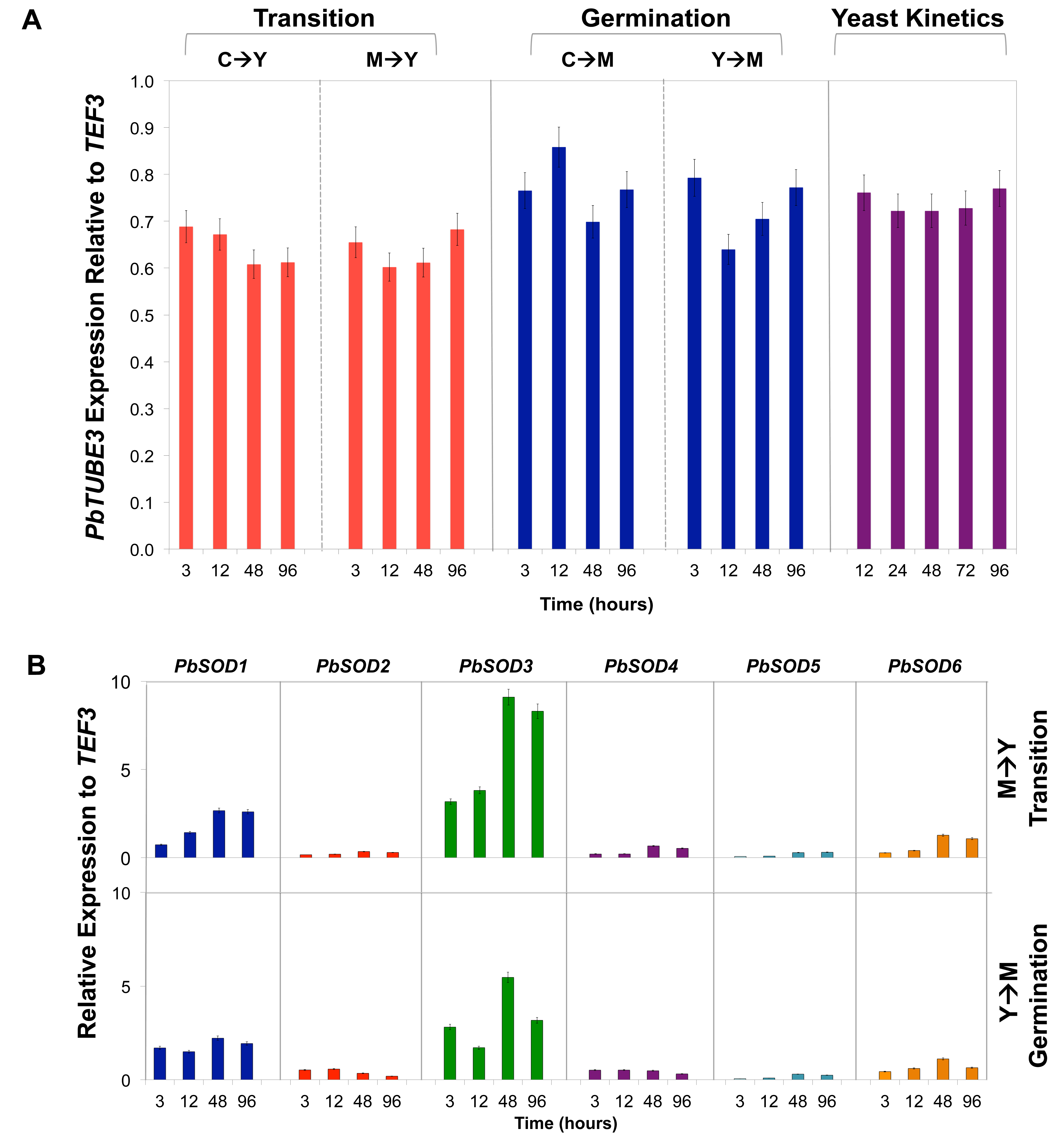

Supplement: S1 Fig — (A) PbTUBE3 gene expression in PbWT60855 cells undergoing morphological switches and during batch culture growth. Gene expression levels were obtained by RT-qPCR assay in order to discard any variation in PbTUBE3 gene expression, confirming in this way that this gene can be used as normalizer under the experimental conditions carried out in this work. The measurement was normalized using as housekeeping gene PbTEF3 in cells undergoing transition (C-Y, M-Y) and germination (C-M, Y-M) processes, and during a batch culture growth. (B) Expression of SOD isoforms during transition from M-Y, and during germination from Y-M. Normalization was performed using TEF3. (TIF) [file pntd.0004481.s002.tif]

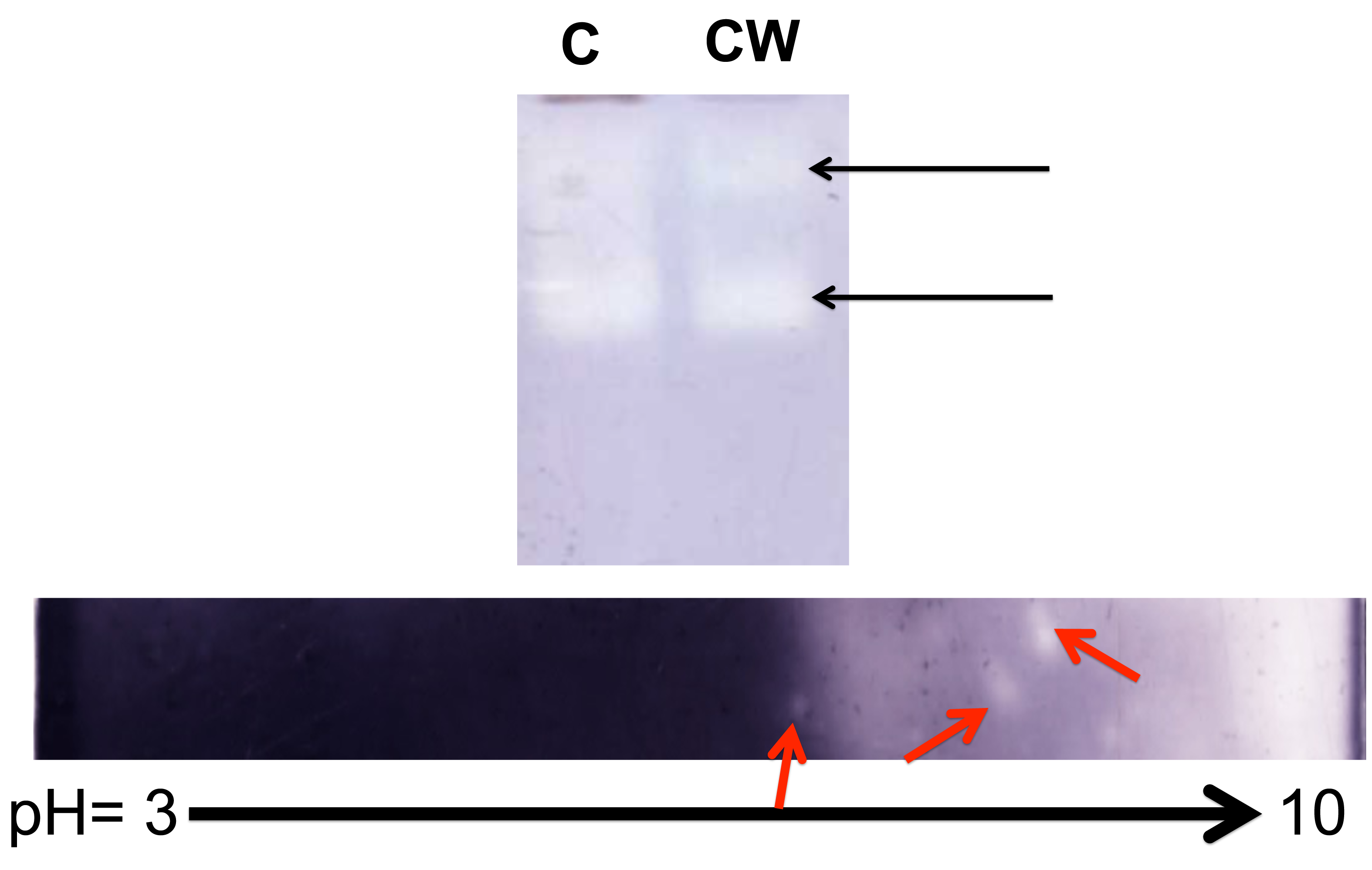

Supplement: S2 Fig — Top: Sod activity in a non-denaturing polyacrylamide gel (1D-PAGE; 150 μg of crude protein extract; C = cytoplasmic fraction, CW = cell wall fraction). In the 1D-PAGE at least two bands can be observed, indicating the Sod activity. In order to obtain a better gel resolution and visualize the six isoforms, a 2D-PAGE was carried out. Bottom: Sod activity in a non-denaturing 2D-PAGE (150 μg of crude protein extract). Illustration shows three spots indicating the activity of Sod isoforms. We were unable to predict to which isoform corresponded every band or spot, but it is clear that they corresponded to the Sods. (TIF) [file pntd.0004481.s003.tif]

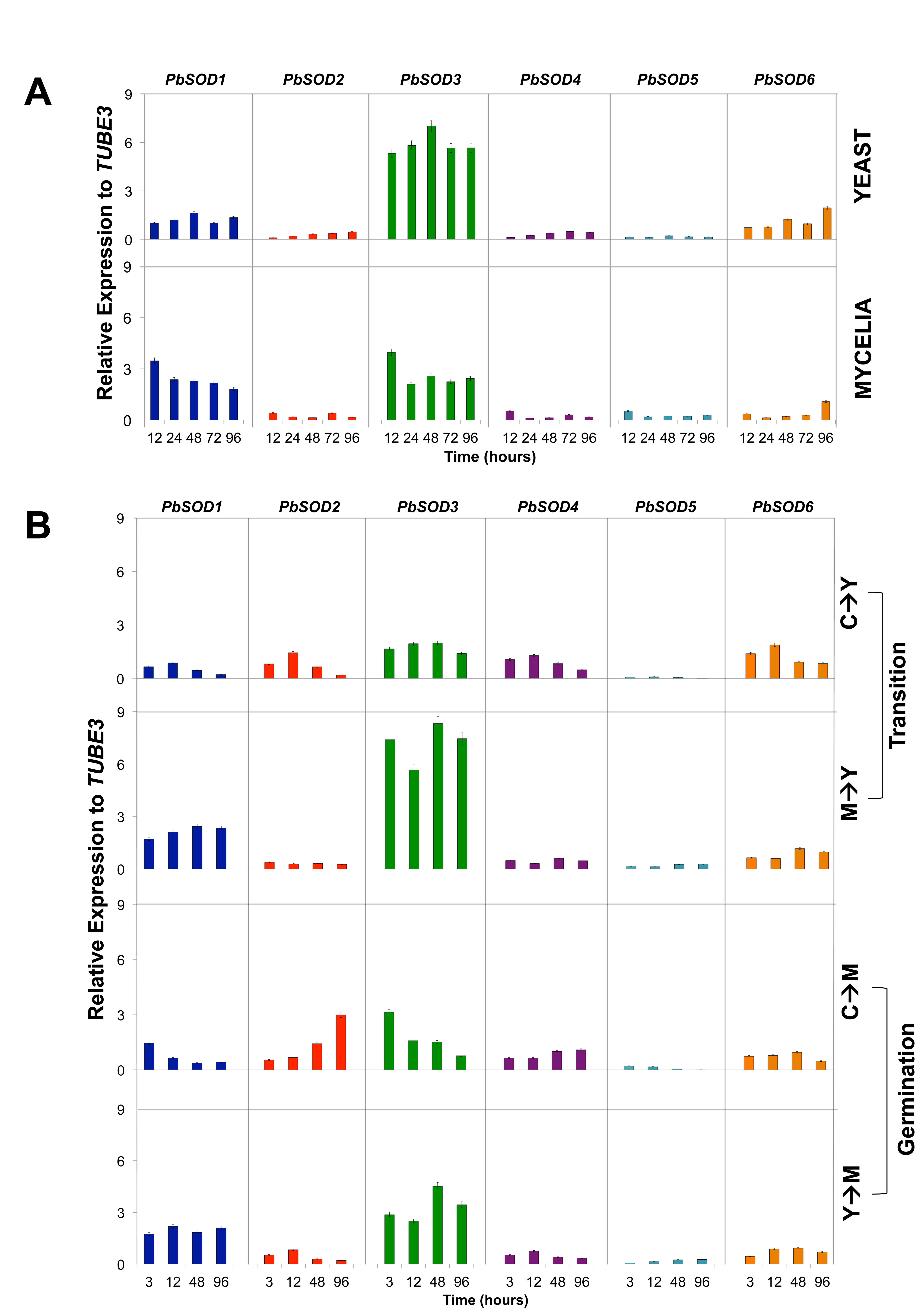

Supplement: S3 Fig — (A) Kinetic expressions during batch culture growth, PbSODs gene expression was evaluated in Pb60855 yeast and mycelia forms. (B) Expression of SOD isoforms during transition from C-Y and from M-Y, and during germination from C-M and from Y-M. Higher expression levels of PbSOD3 during all evaluated events can be observed. During the transition from C-Y and germination from C-M, the expression level of all isoforms was quite similar. On the other hand, levels of SOD1 and SOD3 became more evident during transition from the M-Y and germination from the Y-M. (TIF) [file pntd.0004481.s004.tif]

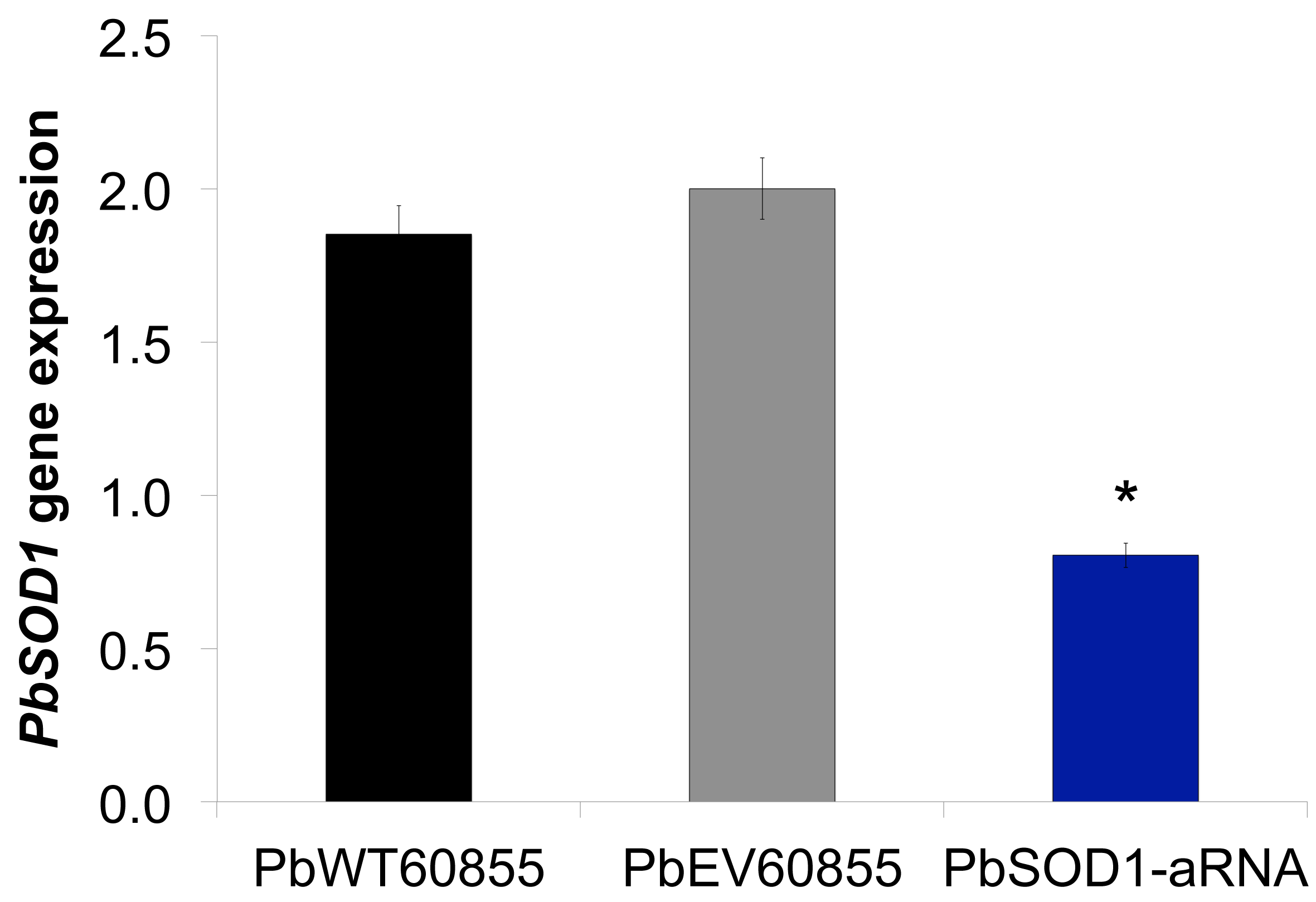

Supplement: S4 Fig — Gene expression levels of PbSOD1 were obtained by RT-qPCR assay. The measurement was normalized with the housekeeping gene β-tubulin in PbWT, PbEV and PbSOD1-aRNA yeast cells grown at exponential phase. Results are the mean of three individual experiments. Asterisks denotes P ≤0.05 compared to PbWT. (TIF) [file pntd.0004481.s005.tif]

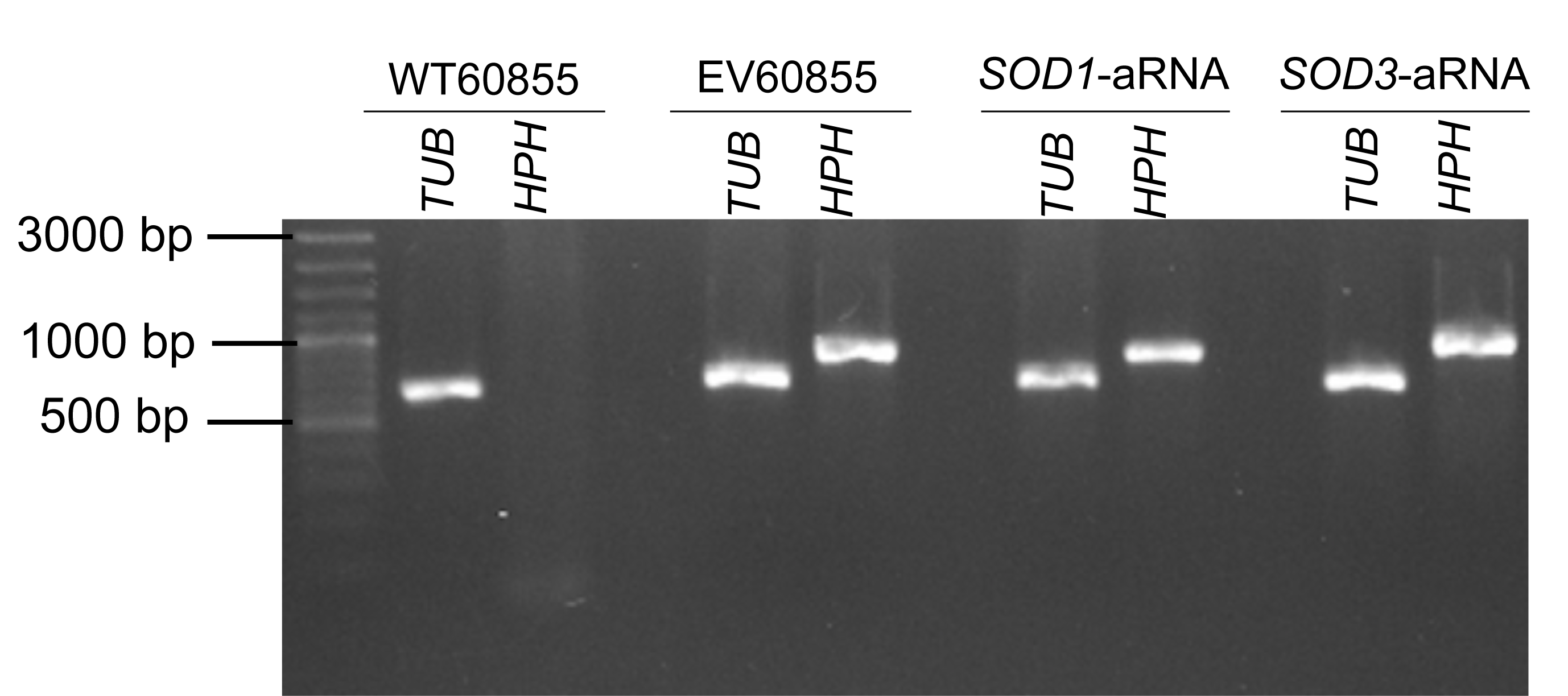

Supplement: S5 Fig — Genomic DNA from the PbWT60855, PbEV60855 and the knockdown strains PbSOD1-aRNA and PbSOD3-aRNA were tested by PCR using specific primers for the tubulin gene (TUB) and for the hygromycin B resistance gene (HPH). (TIF) [file pntd.0004481.s006.tif]

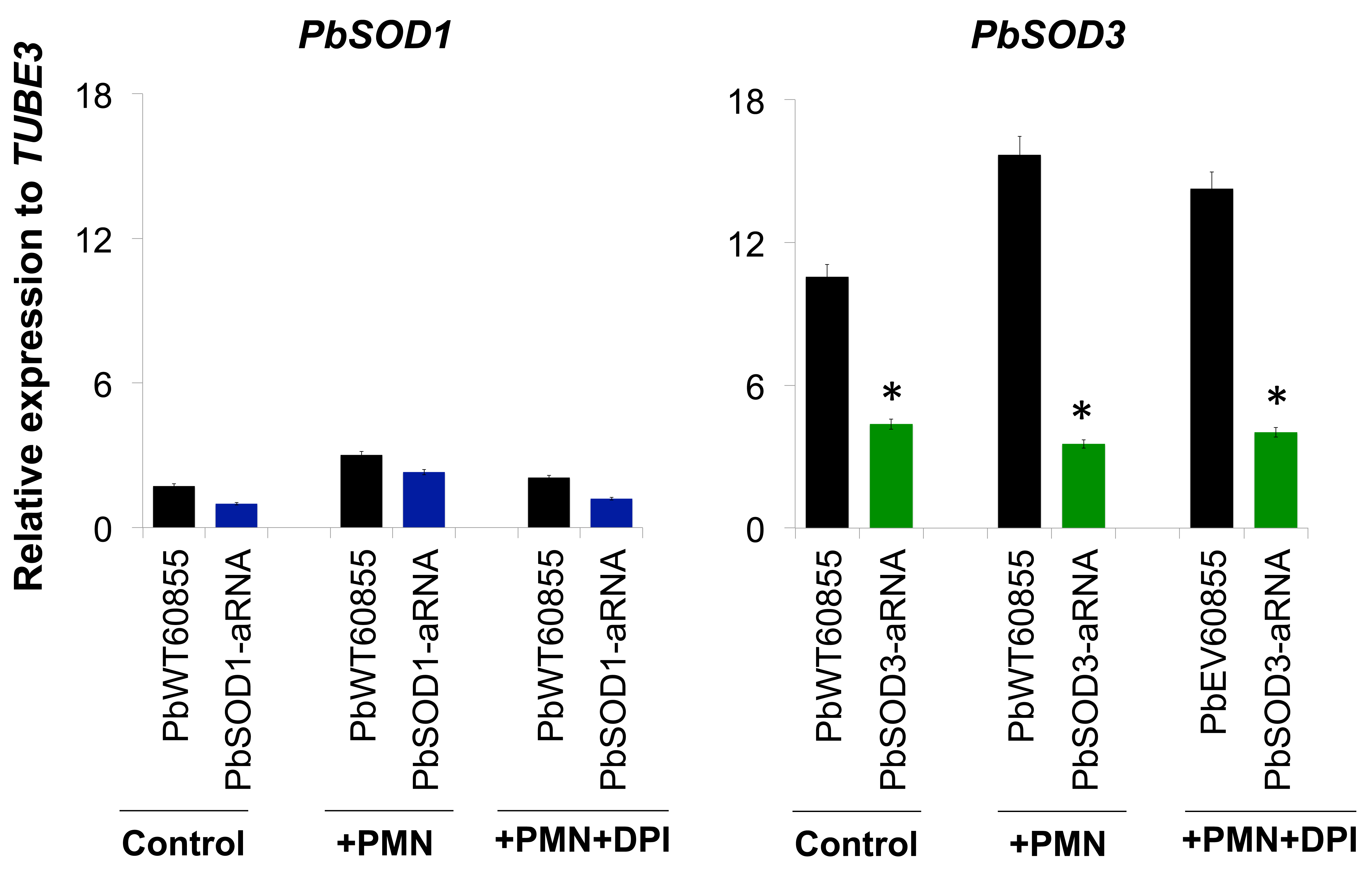

Supplement: S6 Fig — PbSOD1 and PbSOD3 gene expression were determined through RT-qPCR assay and normalized with the housekeeping gene β-tubulin. Results are the mean of three individual experiments. Asterisks denotes P ≤0.05 compared to PbWT. (TIF) [file pntd.0004481.s007.tif]

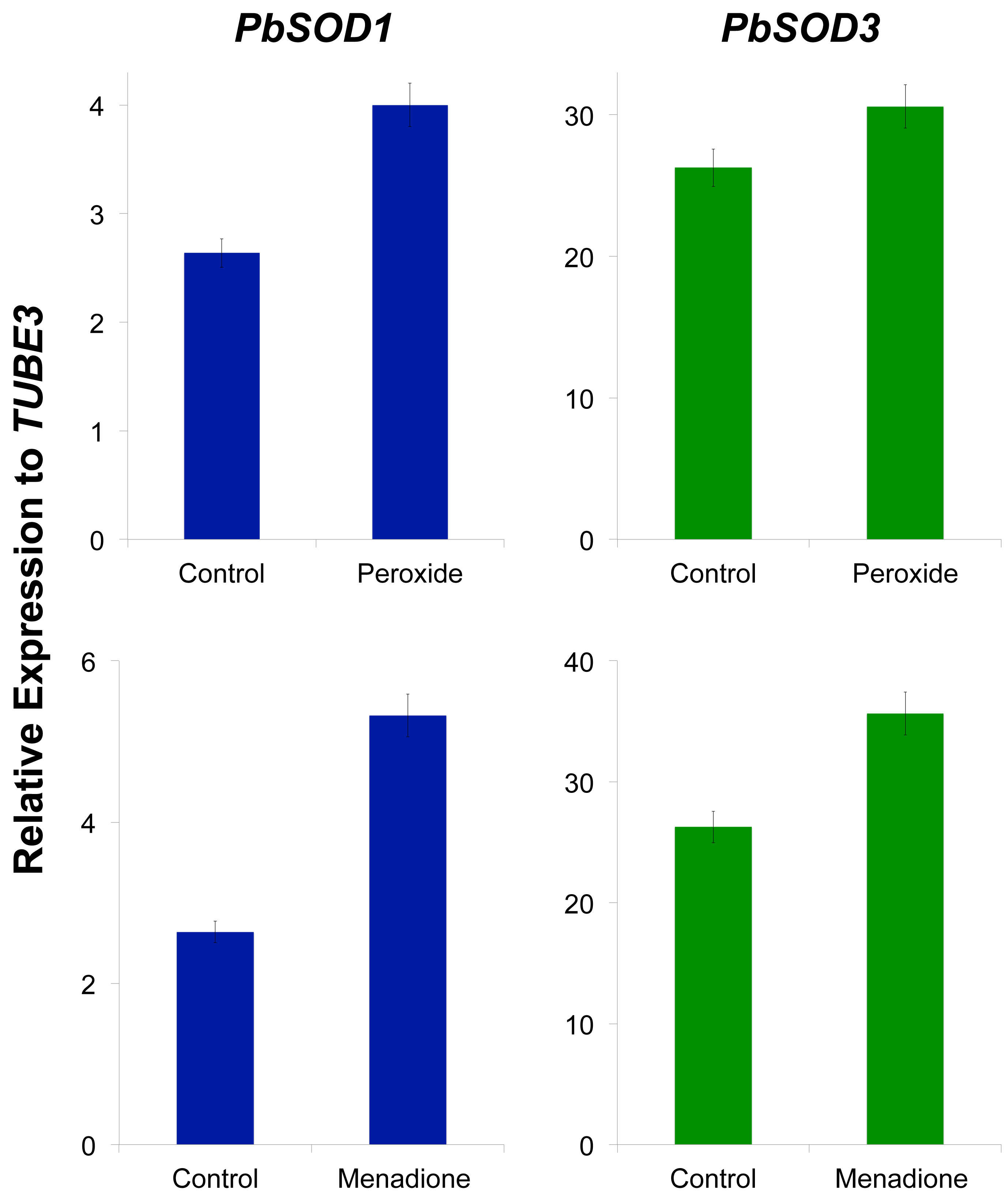

Supplement: S7 Fig — PbSOD1 and PbSOD3 gene expression were determined through RT-qPCR assay and normalized with the housekeeping gene β-tubulin. Results are the mean of three individual experiments. (TIF) [file pntd.0004481.s008.tif]

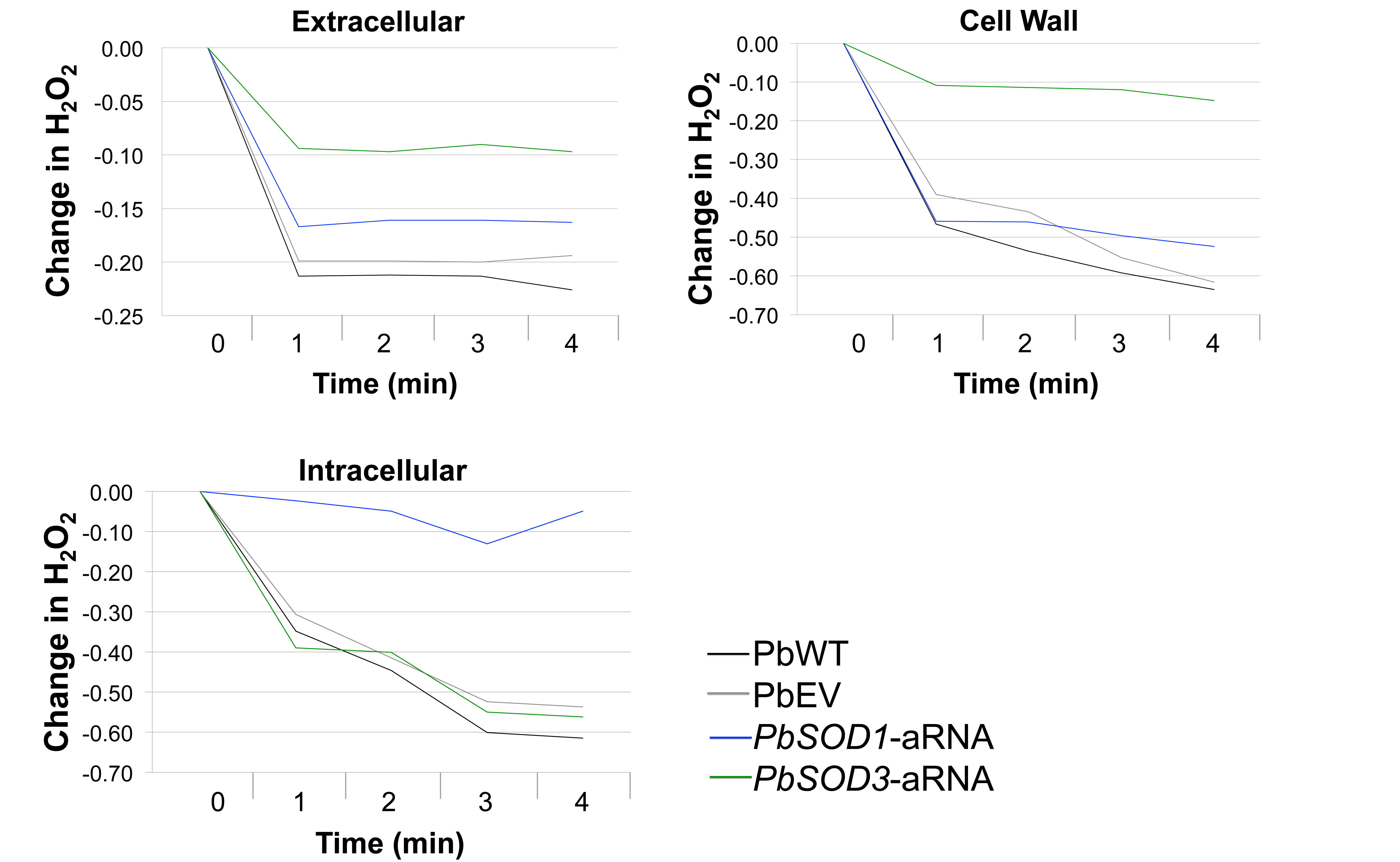

Supplement: S8 Fig — Extracellular, cell wall and cytoplasmic fractions of PbWT, PbEV, PbSOD1-aRNA and PbSOD3-aRNA strains were collected. Relative H2O2 destruction was measured as the decrease in the absorbance at 240 nm. Results are the mean of three individual experiments. (TIF) [file pntd.0004481.s009.tif]
